# Distinct effects of epirubicin, cisplatin and cyclophosphamide on ovarian somatic cells of prepuberal ovaries

**DOI:** 10.18632/aging.102476

**Published:** 2019-11-11

**Authors:** Serena Marcozzi, Valerio Rossi, Giulia Salvatore, Francesca Di Rella, Massimo De Felici, Francesca Gioia Klinger

**Affiliations:** 1 Department of Biomedicine and Prevention, Section of Histology and Embryology, Faculty of Medicine and Surgery, University of Rome Tor Vergata, Rome, Italy; 2 Medical Oncology, Department of Senology, National Cancer Institute, IRCCS Foundation G. Pascale, Naples, Italy

**Keywords:** epirubicin, cisplatin, cyclophosphamide, ovary, chemotherapy

## Abstract

*In vitro* culture models were used to characterize the effects of chemotherapeutic drugs and of LH on somatic cells from prepuberal mouse ovaries. All cell types (pre- and granulosa cells, pre-thecal and OSE cells) underwent apoptosis following Epirubicin (0.5μM) exposure for 24hrs (about 60%) and 48hrs (>80%). Cisplatin (10μM) and the Cyclophosphamide active metabolite, Phosphoramide Mustard (10μM), didn’t cause apoptosis in 90% of pre-thecal and pre-granulosa cells up to 72hrs of exposure, although they suffered extensive DNA damage and cell cycle arrest, and acquired stress induced premature senescence (SIPS) features. Cultured granulosa cells didn’t show evident DNA damage and remained viable without acquiring SIPS features; OSE cells were resistant to apoptosis and SIPS but not to DNA damage. These latter, like pre-thecal and pre-granulosa cells, were able of efficient DNA repair involving MLH1-dependent MMR pathways. SIPS features were also observed in ovary after *in vivo* treatment with Cisplatin. LH (200mIU/mL) didn’t significantly influence apoptosis, SIPS and DNA damage but favoured DNA repair. These results show that somatic cells of prepuberal ovary response to drugs in different ways, either undergoing apoptosis or SIPS, either showing resistance to Cisplatin and Phosphoramide Mustard. Moreover, a new role of LH in promoting DNA repair was shown.

## INTRODUCTION

Ovarian reserve exhaustion causes menopause, a phenomenon that occurs in women of an average age of 50 years. Instead, the prevalence of menopause before the age of 40 years is about 1%, of 0.1% at 30 years of age and uncommon during adolescence [1, 2]. This physiological phenomenon is the result of an ovary incapability to regularly produce new oocytes after birth. Indeed, all the oocytes individually enclosed in primordial follicles (PMFs) present in the ovary during all women postnatal age, are widely considered to be generated only during the embryonic stage, giving rise to a basically non-renewable stockpile termed ovarian reserve.

Under normal conditions, approximately 1–2 million PMFs are present at birth within woman’s ovaries. Among these, only around 400 follicles will reach the pre-ovulatory stage and undergo ovulation, releasing an oocyte available for fertilization. The remaining ~99% of the follicles are progressively fated to undergo programmed cell death, the so-called atresia, without producing a fertilizable oocyte [3–7]. Wallace and Kelsey [7], mathematically estimated that in the majority of 30 year old women is present only 12% of their maximum pre-birth number of PMFs and that this percentage decline to 3% at the age of 40. This process continues until fewer than a thousand follicles remain in the ovary defining the beginning of the menopause [7, 8]. However, it is now well recognized that, under certain circumstances, this process can be deregulated leading to an acceleration of the ovarian reserve exhaustion, a phenomenon collectively known as Premature Ovarian Insufficiency (POI) [9].

Emerging evidences suggests that also iatrogenic agents, such as anticancer regimen (radio and chemotherapy), would be able to reduce fertility in premenopausal women damaging PMFs stockpile. Advancements in early diagnoses and new treatments have greatly increased the survival rate of women with cancer, with a five-year survival rate of 69% in women, value that increase considering only younger patients (15–44 years old) [10]. However, chemo- and radiotherapy can cause long term side effects such as gonad toxicity: in women, therapeutic treatments are able to induce a partial or total reduction of ovarian reserve and premature menopause and infertility [11]. The effects of early menopause may be important, especially at younger biologic ages, with rapid and substantial effects not only on withdrawal of sex hormones and possibility of absence of menarche in pre-pubertal patients, but also on lipid and carbohydrate metabolism [12–14]. For these reasons, in order to guarantee a good quality of future life to survivors, there is a growing interest in the prevention of gonadotoxicity. In the last twenty years, many studies have been focused on the identification of substances that given earlier or together with chemotherapy, protects ovaries from gonadotoxicity [15, 16].

In line with this, our group recently demonstrated the protective effect of Luteinizing Hormone (LH) against ovotoxicity induced by Cisplatin on the ovary of prepuberal mice, both in vitro and *in vivo*. The finding of a subset of ovarian somatic cells expressing the receptor for LH in these ovaries, suggested that the protection exerted by the hormone was indirect and mediated by these somatic cells [17].

The primary aims of the present work are, therefore, to characterize the effects of three common chemotherapeutic drugs, Cisplatin (CS) and Epirubicin (EPI) and Cyclophosphamide (CPM) on the somatic cells of the prepuberal mouse ovaries, to identify their target cells, the action mechanism of such drugs and how LH could interfere with such mechanisms

## RESULTS

### Characterization of the ovarian somatic cell populations in culture

**Figure 1:**
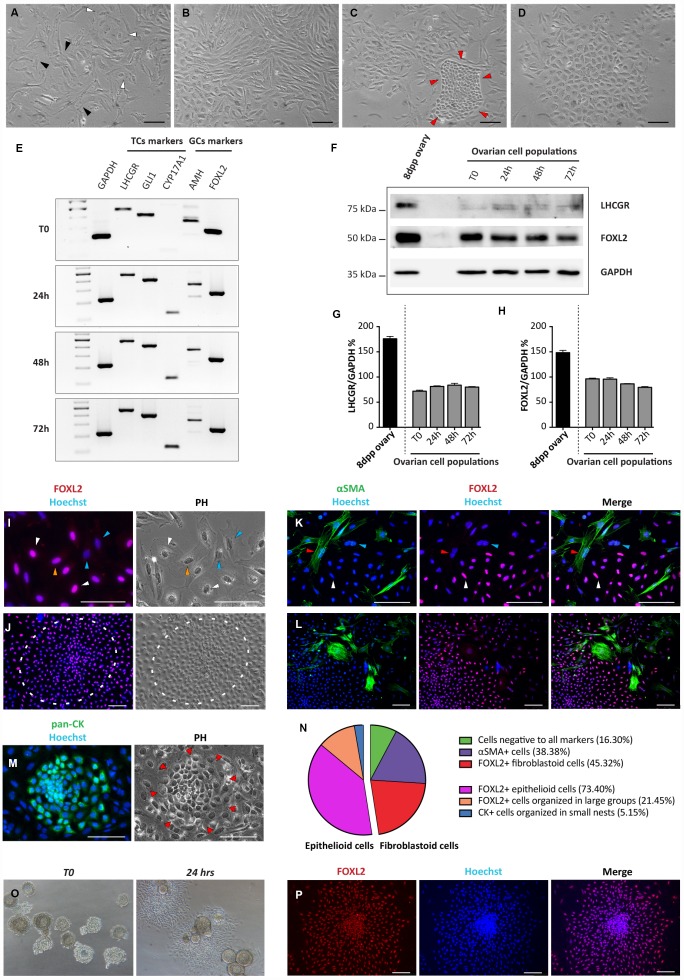
**Morphological and molecular characterization of cultured ovarian somatic cells.** (**A**–**D**) Phase contrast (PH) observations after 24 hrs of culture. (**A**) White and black arrowheads indicate scattered epithelioid and fibroblastoid cells, respectively. (**B**) Fibroblastoid cells at higher density. (**C**) A little colony of small epithelioid cells (red arrowheads) surrounded by fibroblastoid cells. (**D**) A large colony of polygonal epithelioid cells. Scale bar = 100 μm. (**E**) RT-PCR analyses for granulosa (AMH and FOXL2) and theca (LHCGR, Gli1 and CYP17A1) cells markers (GAPDH was used as housekeeping gene). (**F**) WB analysis for LHCGR and FOXL2 after increasing culture times. (GAPDH was used as housekeeping protein). (**G**, **H**) Densitometric quantification of proteins relative expression is reported. Data are expressed as mean ± SEM of three analyses. (**I**-**M**) Representative field of IF for FOXL2, αSMA and pan-CK after 24 hrs of culture, scale bar = 100 μm. (**I**, **J**) FOXL2 identifies cells with different morphologies: (**I**) scattered epithelioid and fibroblastoid cells (white and yellow arrowheads, respectively); blue arrowheads pointed fibroblastoid FOXL2 negative cells and (**J**) a large colony of polygonal epithelioid cells (white circle). (**K**, **L**) Double IF for αSMA and FOXL2. Cells positive for αSMA showed a fibroblastoid morphology. Note that IF for αSMA and FOXL2 never overlappe3d (red arrowheads indicate αSMA positive and FOXL2 negative cells, white arrowheads FOXL2 positive and αSMA negative cells; blue arrowheads indicate a double negative cell). (**M**) Little colony of pan-CK positive small epithelioid cells. (**N**) Quantification of the FOXL2, αSMA and pan-CK positive and triple-negative cells. (**O**, **P**) Follicles isolated from 16dpp mouse ovaries cultured for 24 hrs. (**O**, **P**) Follicular cells, spread out to form an epithelioid cell monolayer, showed FOXL2 (red) positivity.

Ovarian somatic cells obtained from 8dpp ovaries were cultured for 4 days under the conditions described in M&M. From 2 up to 4 days of culture, a mixture of four distinct cell types was observed under a phase contrast (PH) microscope. For the most part, cells were a mash-up of loose confluent fibroblastoid cells with multipolar shape mixed with cells showing a flattened polygonal shape typical of epithelial cells (Figure 1A, 1B). However, colonies of compact small size cells or large epithelioid cells with small nucleus were also detected (Figure 1C, 1D).

Cells were analyzed by RT-PCR, WB, IHC and IF, at different culture times (between day 1 and day 4) for the expression of markers of epithelial, granulosa (GCs) and follicular theca (TCs) cells (pan-CKs; AMH and FOXL2; Gli1, LHCGR, CYP17A1 and αSMA, respectively).

RT-PCR analyses showed that the transcripts of all these proteins were expressed at significant levels by the cultured cells at T0 (Figure 1E). The expression of FOXL2 and LHCGR, was confirmed at protein level by WB (Figure 1F–1H). Except for increasing level of CYP17A1 (a marker of steroidogenic TCs), no significant changes in the expression of all other markers during the culture period were observed (Figure 1E–1H).

IHC and IF showed that the about 40%–50% of the fibroblastoid cells and almost all dispersed and in colonies epithelioid cells, were positive for FOXL2. We considered such scattered cells as stromal precursor granulosa cells (pGCs), and the large epithelioid cells in compact colonies as granulosa cells (GCs), based on FOXL2 positivity, morphologies and on published observations. In fact, in early postnatal mouse ovaries it was previously described the presence of granulosa cells from follicles, activated immediately after birth and of a subset of stromal cells representing the precursors of granulosa cells that populate the cortical primordial follicles forming at this stage [18]. Of the remaining fibroblastoid cells about 35%–45% were αSMA positive (possibly pre-thecal cells, pTC) [19] while the rest (5%–15%) appeared negative for both markers. Double IF for FOXL2 and αSMA showed that these markers never overlapped. Finally, pan-CK positive cells corresponded to the epithelioid cells in small colonies (likely ovarian surface epithelium, OSE, cells) (Figure 1I–1N). As note, the percentage of the cells positive to these markers remained substantially unchanged during the culture time (data not shown).

IF on ovary sections from 8dpp mice confirmed the specificity of the FOXL2 and αSMA markers showing that only GCs of the various follicle types were FOXL2 positive, whereas αSMA was confined to the TCs layers of growing follicles (Supplementary Figure 1A, 1B). Similarly, GCs spreading out from *in vitro* cultured oocyte-free secondary follicles obtained from 16dpp mice showed morphological features and FOXL2 positivity like putative GCs (Figure 1O, 1P).

**Figure 2:**
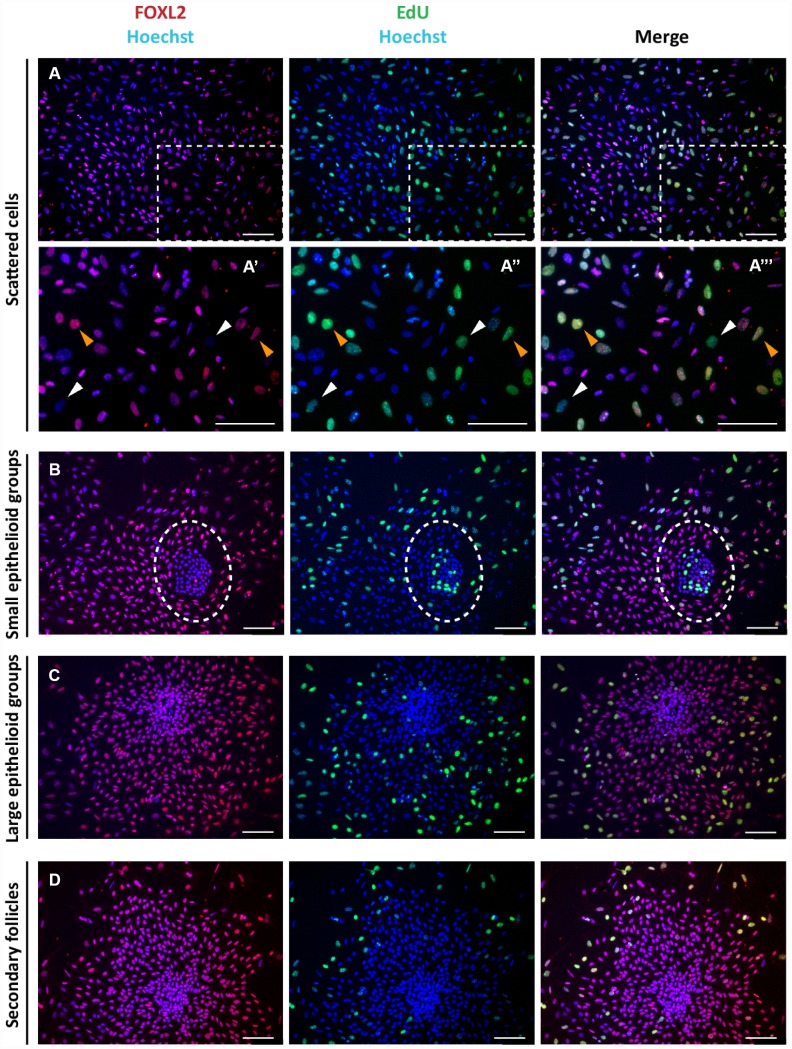
**Analysis of proliferation state of cells in culture.** Representative double staining for Click-iT EdU (green) and FOXL2 (red) on cultured cells (**A**–**C**) and isolated secondary follicles (**D**) after 24 hrs of culture. Orange and white arrowheads indicate proliferating FOXL2 positive and negative cells, respectively (**A’**-**A’’’** higher magnification images from **A**). © GCs in large colonies and (**D**) GCs spreading out from secondary follicles were negative for Click-iT EdU proliferation assay. Scale bar = 100μm.

The Click-iT EdU proliferation assay performed on the cultured cells indicated that for the most part, the scattered putative pGCs, pTCs and OSE cells in colonies were proliferating, whilst GCs in large colonies and spreading out from secondary follicles were not (Figure 2).

### Epirubicin induces apoptosis and extensive DNA damage in all ovarian somatic cells

In order to characterize the EPI effect on ovarian somatic cells, the cell cultures were exposed to 0.5 μM EPI (corresponding to about 0.3 μg/mL), a concentration in the high therapeutic range [20].

**Figure 3:**
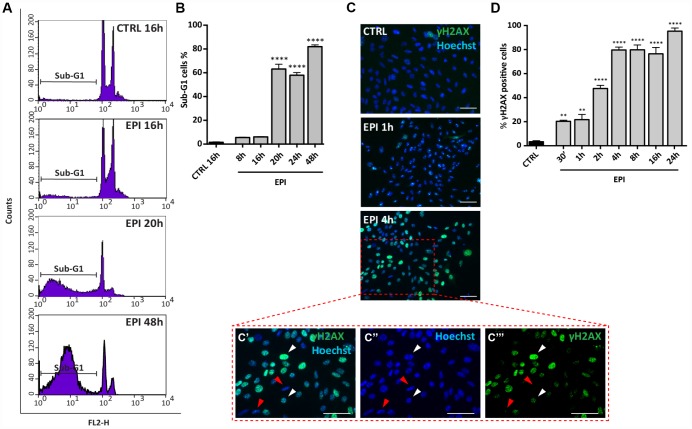
**Analysis of EPI-induced apoptosis in ovarian somatic cells.** (**A**, **B**) Cells treated with 0.5 μm EPI for the indicated times were analyzed by flow cytometry, sub-G1 phase represents apoptotic cells. Data are expressed as mean ± SEM of three different experiments. Statistical differences *vs* control ****p<0.0001. © Representative IF for γH2AX in the same cells at the indicated times, scale bar = 50 μm. (**C’**–**C’’’** higher magnification images from **C**). White and red arrowheads indicate γH2AX positive and negative cells, respectively. (**D**) The graph reports the quantification of γH2AX positive cells percentage scored in three different experiments. Data are expressed as mean ± SEM. Statistical differences *vs* control **p<0.01 ****p<0.0001.

Propidium Iodide (PI) cells fluorescence, evaluated by flow cytometry, after 8 to 48 hrs of culture, indicated that, while in the control group the percentage of cells in sub-G1 phase (considered apoptotic cells) remained stable (1.46 ± 0.34%), it increased significantly in the presence of EPI from 16 hrs (6.1 ± 0.2%) onwards and reached 63.16 ± 4.05% at 20-24 hrs and 82.03 ± 1.52% at 48 hrs (Figure 3A, 3B).

*In situ* IF for the phosphorylated form of H2AX (γH2AX), a marker of DNA damage, showed that EPI caused a progressive rapid increase of the positive cells number, reaching 80% after 4 hrs of culture (CTRL = 3.3 ± 0.9% *vs* EPI 4h = 79.7 ± 2.4%) and maintained up to 95.33 ± 2.60% after 24 hrs (Figure 3C, 3D). These last results were confirmed by WB analyses (Supplementary Figure 2).

### Cisplatin does not induce apoptosis in the ovarian somatic cells but causes stress-induced premature senescent in putative pGCs and pTCs

In order to analyze the effect of CS on ovarian cell populations, cultures were exposed to 10 μM CS (corresponding to about 3 μg/mL) up to 72 hrs. This concentration was chosen on the basis of our previous results [17], in the high therapeutic range [21, 22].

**Figure 4:**
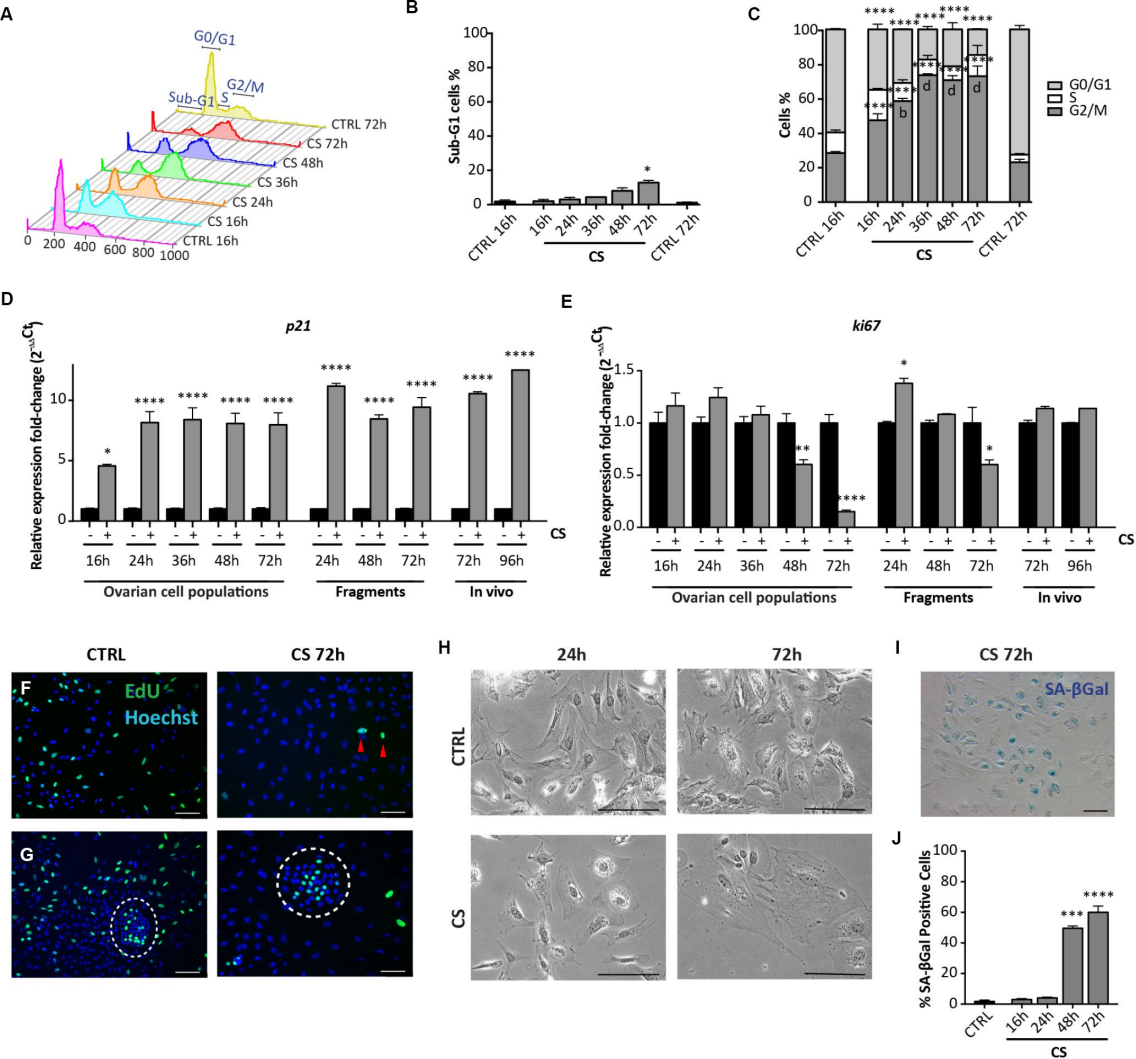
**Analysis of CS-induced senescence in ovarian somatic cells *in vitro* and *in vivo*.** (**A**–**C**) Cells treated with 10 μM CS for the indicated times, were analyzed by flow cytometry in order to quantify (**C**) cell cycle distribution and (**B**) apoptosis (sub-G1). Results are expressed as mean ± SEM of three experiments. Statistical differences *vs* control *p<0.05 ****p<0.0001 Statistical differences *vs* 16hrs in the G2/M phase *b* = p<0.01 *d* = p<0.0001. No significant differences were observed between 36, 48 and 72 hrs in the G2/M phase. (**D**, **E**) Comparison of qRT-PCR analysis for (**D**) p21 and (**E**) ki67 between *in vitro* (dispersed ovarian somatic cells and ovarian fragments) and *in vivo* (ovaries from intraperitoneal injected mice) conditions. Data are shown as mean ± SEM of three analyses. Statistical differences *vs* control *p<0.05 ****p<0.0001. (**F**, **G**) Representative staining for Click-iT EdU (green) in (**F**) scattered cells and in (**G**) colony of ovarian surface epithelium (OSE) cells (white circle) after 72 hrs of CS treatment. Red arrowheads indicate proliferating cells. Scale bar = 100μm. (**H**) Cultured ovarian somatic cells treated with CS acquired large and flattened morphology from 48 hrs onwards. (**I**, **J**) Representative image and quantification of SA-βgal activity positive cells after 72 hrs of CS treatment, scale bar = 100μm. Data are expressed as mean ± SEM of four experiments. Statistical differences *vs* control ***p<0.001 ****p<0.0001.

Flow cytometric analyses showed that, differently from EPI, CS caused only a slight increase of the percentage of apoptotic cells both after 48 hrs (CTRL = 1.46 ± 0.34% *vs* CS = 8.05 ± 1.29%), and 72 hrs (CTRL = 1.46 ± 0.34% *vs* CS = 12.85 ± 0.98%) of culture. At the same time, it was evident that CS treatment resulted in a progressive accumulation of cells in G2/M stage that peaked at 36 hrs (16 hrs: 47.32 ± 1.43%; 24 hrs: 58.51 ± 1.53%; 36 hrs: 73.45 ± 0.53%; 72 hrs: 72.92 ± 5.85%) (Figure 4A–4C). Such cell cycle arrest was confirmed by a marked decrease of the *Ki67* (about 7-fold) and increase of *p21* (about 8-fold) transcripts during the same time frame (Figure 4D, 4E) and by the Click-iT EdU proliferation assay (Figure 4F, 4G). Surprisingly, this assay showed that the majority of putative OSE cells continued to proliferate in spite of CS treatment (Figure 4G).

Observations under the PH microscope showed that, between 48 and 72 hrs of CS treatment, 60%–70% of the putative pGCs and pTCs acquired a large and flattened morphology, typical of stress-induced premature senescent (SIPS) cells (Figure 4H). Such morphological change was not evident in the putative GCs and OSE cells, these latter colonies showing appreciable size increase (not shown).

We also found that virtually all cells with large and flat morphologies following CS exposure, acquired high activity for the senescent associated βgal (SA-βgal), a typical feature of senescent cells [23] (Figure 4I, 4J). Conversely, putative GCs and OSE cells were SA-βgal positive both in the control and Cs-treated conditions (not shown).

Taken together these results indicated that the actively proliferating putative pTC and pGCs, present in our cultures, reacted to CS acquiring SIPS features rather than undergoing apoptosis, while putative GCs were resistant to the drug probably because contact inhibited. On the other hand, putative OSE cells were CS resistant despite active proliferation.

**Figure 5:**
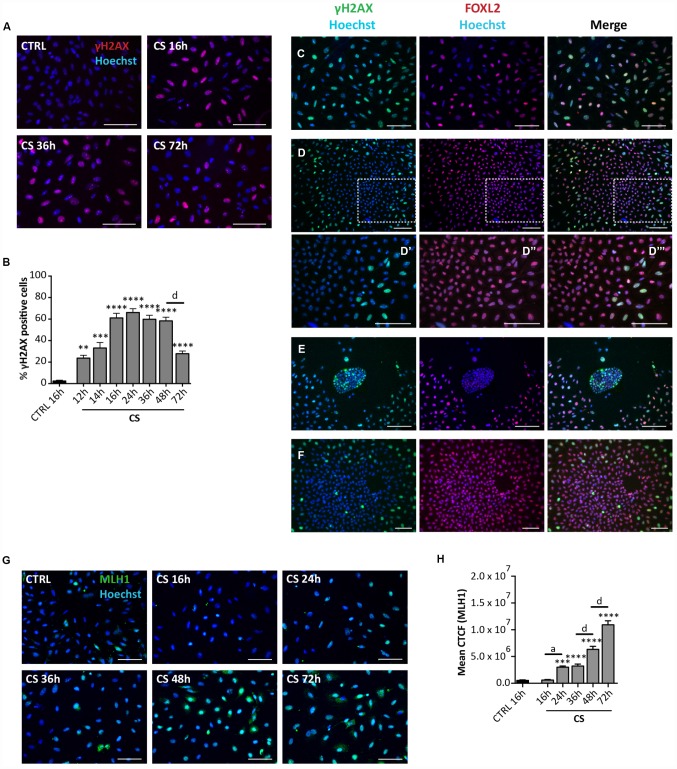
**Analysis of CS-induced DNA damage in ovarian somatic cells *in vitro*.** (**A**) Representative IF of cells stained with γH_2_AX (red) after 16, 36 and 72 hrs of treatment with 10 μM CS. (**B**) The graph reports the quantification of γH2AX positive cells percentage. Data are shown as mean ± SEM of three experiments. Statistical differences *vs* control **p<0.01 ***p<0.001 ****p<0.0001; CS 48 hrs *vs* CS 72 hrs *d* = p<0.0001. Note that cells showed a progressive increase in percent of positive cells that peaked after 16 hrs followed by a marked decrease at 72 hrs. (**C**–**F**) Representative double IF for γH2AX (green) and FOXL2 (red) in (**C**) scattered fibroblastoid and epithelioid cells, (**D**) a large epithelioid colony (**D’**–**D’’’** higher magnifications from **D**), (**E**) a little colony of small epithelioid cells and (**F**) GCs from isolated secondary follicles after CS-treatment for 24 hrs. Scale bar = 100μm. (**G**) Representative IF of cells stained with MLH1 (green) after 16, 24, 36, 48 and 72 hrs of treatment with 10 μM CS. Scale bar = 100 μm (**H**) The graph reports the Mean Correlated Total Cell Fluorescence (CTCF) in ovarian cells treated with/out CS as indicated. The fluorescence intensity was determined in each cell by ImageJ software. Data are expressed as means ± SEM of three experiments. Statistical differences *vs* control ***p<0.001 ****p<0.0001; CS 16 hrs *vs* CS 24 hrs *a =* p<0.05; CS 36 hrs *vs* CS 48 hrs *d* = p<0.0001; CS 48 hrs *vs* CS 72 hrs *d* = p<0.0001. Note that cells showed a progressive increase in the nuclear expression of MLH1.

Since SIPS usually takes place following the activation of the DNA damage response (DDR) pathways, we next investigate the incidence of DNA damage in the cultured cells. IF staining for γH2AX showed a progressive increase of the number of positive cells up to about 60% of the total cells at 16 hrs (60.95 ± 4.22%) and 48 hrs (58.21 ± 3.31%) from the CS exposure followed by a rapid decrease to 27.73 ± 2.37% at 72 hrs (Figure 5A, 5B). It is likely that such a decrease testifies ongoing DNA repair since not increased apoptotic level was detected at this time (see the results above). The changes of γH2AX amount in the cultured cells detected by WB follow a similar pattern (Supplementary Figure 3A, 3B). The in situ staining pattern of MLH1 (a protein involved in the DNA mismatch repair system (MMR), showing the most part of cells positive and a progressive increase of staining intensity up to 72 hrs (Figure 5G, 5H), on one side confirmed the kinetics of DNA repair reported above for γH2AX and the other suggested the involved of MMR pathways in this process. Cells stained with antibodies against RAD51 and ERCC1 implicated in homologous recombination (HR) and nucleotide excision repair (NER), respectively, showed a generalized constant low/average positivity throughout the tested period that did not allowed any certain conclusion (Supplementary Figure 4A, 4B).

Interestingly, the γH2AX staining kinetics observed *in situ* occurred in all cultured cells except in putative CGs. In fact, these latter showed a constant background level of γH2AX staining like control (24 hrs: % γH2AX-positive scattered cells = 73.90 ± 1.17%; % γH2AX-positive OSE cells = 63.2 ± 0.71%; % γH2AX-positive GCs = 1.4 ± 0.16%) (Figure 5C–5E), suggesting resistance to CS-induced DNA damage. GCs spreading out from *in vitro* cultured oocyte-free secondary follicles treated with CS showed background γH2AX staining like putative GCs (24 hrs: % γH2AX-positive GCs from secondary follicles = 1.81 ± 0.20%) (Figure 5F).

### Cisplatin induces markers of SIPS in cultured ovarian fragments and ovaries *in vivo*

To exclude that the senescence response by the ovarian cells to CS was due to the tissue disaggregation and/or to the in vitro conditions, we next analyzed the expression of stress-senescent markers in cultured CS-treated fragments of ovaries and in ovaries dissected from *in vivo* CS- treated mice. For these analyses, we could employ only qRT-PCR for *p21* and *ki67*, because detection of morphological changes and staining for SA-βgal activity resulted unreliable both in the ovarian fragments and whole ovaries. The results showed that in ovarian fragments exposed to CS for 72 hrs, the levels of ki67 mRNA were significantly reduced about 2-fold and that of p21 mRNA increased about 9-fold in comparison to control (Figure 4D, 4E). A similar up regulation of the p21 transcripts in comparison to control was observed in ovaries after 72 hrs and 96 hrs from CS injection, whereas the expression of ki67 did not change significantly (Figure 4D, 4E).

### The effects of cyclophosphamide on the ovarian somatic cells are similar to that caused by cisplatin

In order to analyze the effect of CPM on ovarian cell populations, cultured cells were exposed to 10 μM of its active metabolite Phosphoramide Mustard (PM), for up to 120 hrs. This concentration (corresponding to about 3 μg/mL) was chosen on the basis of previous studies performed on the mouse ovaries [24], in the range of CPM therapeutic dosage [25].

**Figure 6:**
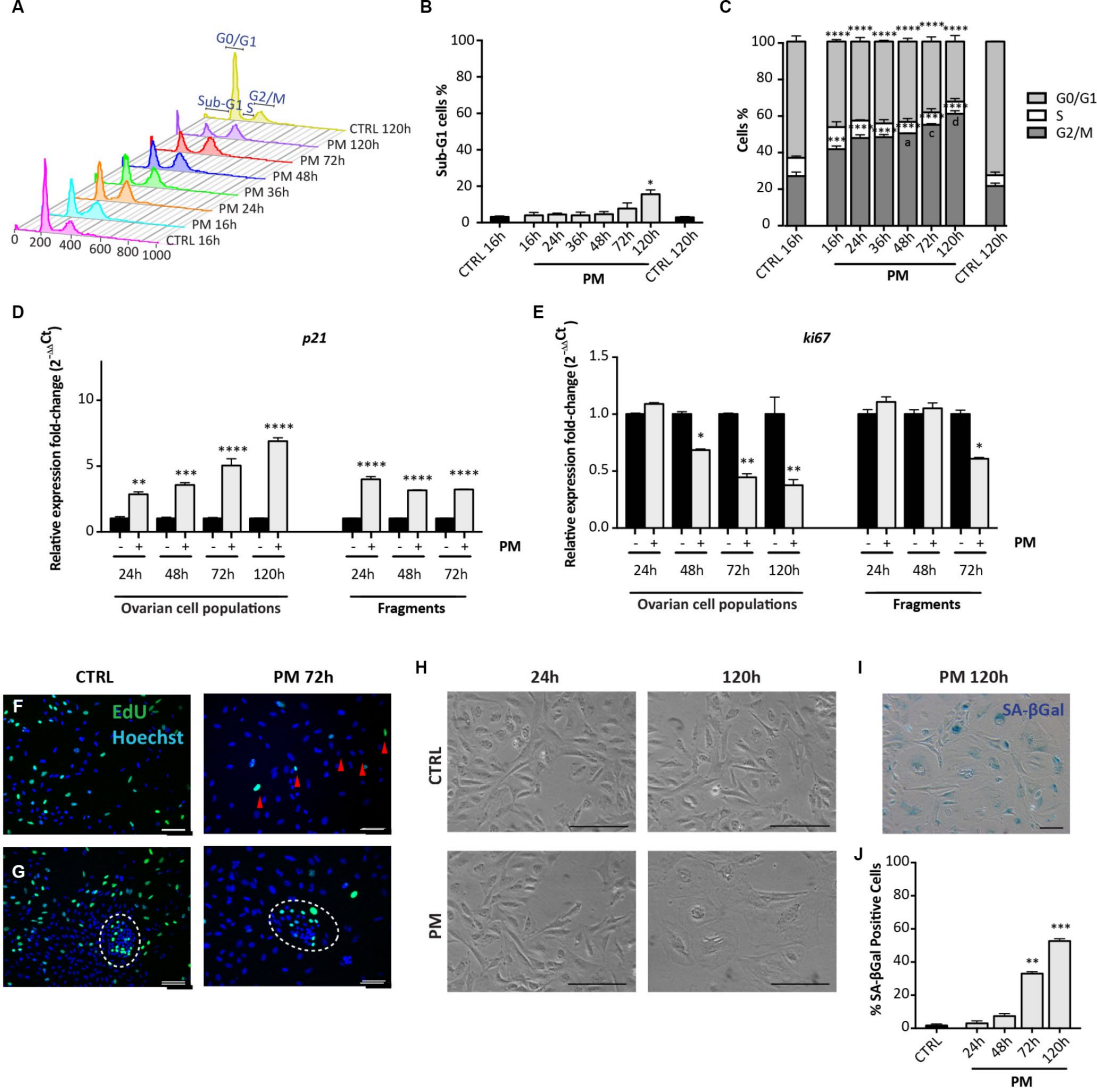
**Analysis of PM-induced senescence in ovarian somatic cells.** (**A**–**C**) Cells treated with 10 μM PM for the indicated time were analyzed by flow cytometry in order to quantify (**C**) cell cycle distribution and (**B**) apoptosis (sub-G1). Results are expressed as mean ± SEM of three experiments. Statistical differences *vs* control **p<0.01 ****p<0.0001. Statistical differences *vs* 16hrs in the G2/M phase *a* = p<0.05 *c* = p<0.001 *d* = p<0.0001. (**D**, **E**) Comparison of qRT-PCR for (d) p21 and (e) ki67 between *in vitro* cultured dispersed ovarian cells and ovarian fragments. Data are shown as mean ± SEM of three analyses. Statistical differences *vs* control *p<0.05 **p<0.01 ***p<0.001 ****p<0.0001. (**F**, **G**) Representative staining for Click-iT EdU (green) in (**F**) scattered cells and in (**G**) little colony (white circle) after 72 hrs of treatment with PM. Red arrowheads indicate proliferating cells. Scale bar = 100μm. (**H**) Cultured ovarian somatic cells acquired large and flattened morphology from 72 hrs of culture with PM. (**I**, **J**) Representative image and quantification of cells positive for SA-βgal activity after 120h of PM treatment. Data are expressed as mean ± SEM of SA-βgal positive cells percentage scored in three experiments. Statistical differences *vs* control **p<0.01 ***p<0.001. Scale bar=100μm.

Flow cytometric analyses showed that similarly to CS but with a slight delay, PM caused a modest increase of the percent of apoptotic cells after 72 hrs (CTRL = 1.46 ± 0.34% *vs* PM = 7.75 ± 2.18%) and 120 hrs (CTRL = 1.46 ± 0.34% *vs* PM = 15.61 ± 1.69%) in comparison to the control (Figure 6A, 6B). Moreover, a progressive arrest of cells in G2/M stage occurred, reaching 60.78 ± 1.68% after 120 hrs from PM addition to the culture medium (Figure 6A, 6C). At this time, cell cycle arrest in the PM-treated cells was confirmed by qRT-PCR analyses for *Ki67* and *p21* genes (Figure 6D, 6E) and the proliferation assay (Figure 6F, 6G).

**Figure 7:**
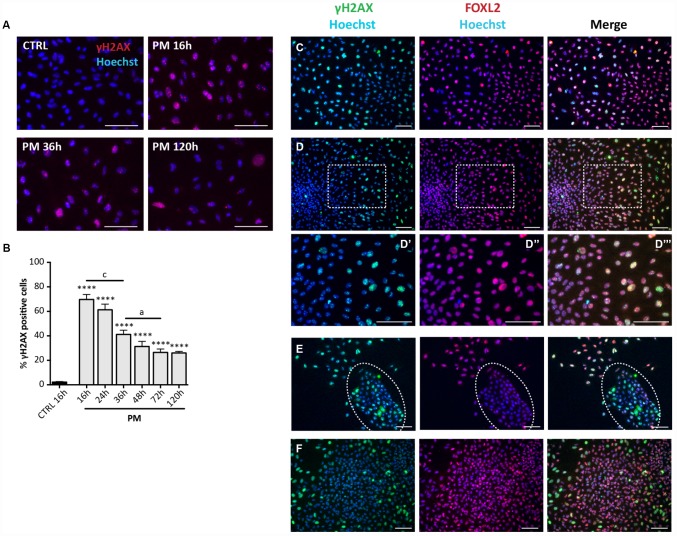
**Analysis of PM-induced DNA damage in ovarian somatic cells.** (**A**) Representative IF for γH2AX in somatic cells treated with PM. (**B**) The graph reports the quantification of γH2AX positive cells percentage. Data are shown as mean ± SEM of three experiments. Statistical differences *vs* control ****p<0.0001. CS 16 hrs *vs* CS 36 hrs *c =* p<0.001; CS 36 hrs *vs* CS 72 hrs *a* = p<0.05. Note an increase in the % of positive cells after 16 hrs of treatment followed by a progressive marked decrease up to 72 hrs. (**C**–**F**) Representative double IF for γH2AX (green) and FOXL2 (red) in (**C**) scattered fibroblastoid and epithelioid cells, (**D**) a large epithelioid colony (**D’**–**D’’’** higher magnification images from **D**), (**E**) a little colony of small epithelioid cells (white circle) and (**F**) GCs from isolated secondary follicles after PM-treatment for 24 hrs. Scale bar = 100μm.

Observations under the PH microscope evidenced PM-induced morphological changes and increased SA-βgal activity in the cultured cells attributable to SIPS and comparable to that induced by CS (Figure 6H, 6J). Also, the pattern of cell positivity to γH2AX in presence of PM, resembled that described for CS, although with faster kinetics in DNA damage and apparent repair (Figure 7A, 7B). As note, like for CS, putative GCs in large colonies and GCs from secondary follicles appeared resistant to the DNA damage detectable by γH2AX staining caused by PM (16 hrs: % γH2AX-positive scattered cells = 76.81 ± 1.16%; % γH2AX-positive OSE cells = 68.05 ± 0.92%; % γH2AX-positive GCs = 1.6 ± 0.23%; % γH2AX-positive GCs from secondary follicles = 2.04 ± 0.20%) (Figure 7C–7F).

Finally, qRT-PCR performed on ovarian tissue fragments showed that after 72 hrs of culture, PM exposure induced about a 4-fold increase of the level of p21 mRNA and a 1.7-fold decreased of the Ki67 mRNA levels in comparison to control (Figure 6D, 6E).

### LH does not protect from EPI-induced apoptosis and does not prevent CS-induced SIPS but favors DNA repair

In previous studies, we reported that 200 mIU/mL LH, through its action on the somatic cells of prepuberal ovaries, protected PMF-enclosed oocytes (POs) from CS-induced apoptosis [17]. On the other hand, here we report that LH was unable to protect these oocytes from EPI-induced apoptosis (% healthy POs 24 hrs: CTRL = 95.24 ± 0.49%; LH = 96.53 ± 0.76%; EPI = 22.7 ± 5.02%; EPI+LH = 24.72 ± 7.10%) (Supplementary Figure 5). The distinct effects of EPI and CS on the somatic cells of prepuberal ovaries reported in the present paper, prompted us to investigate whether the hormone interfered with some of the drug effects.

**Figure 8:**
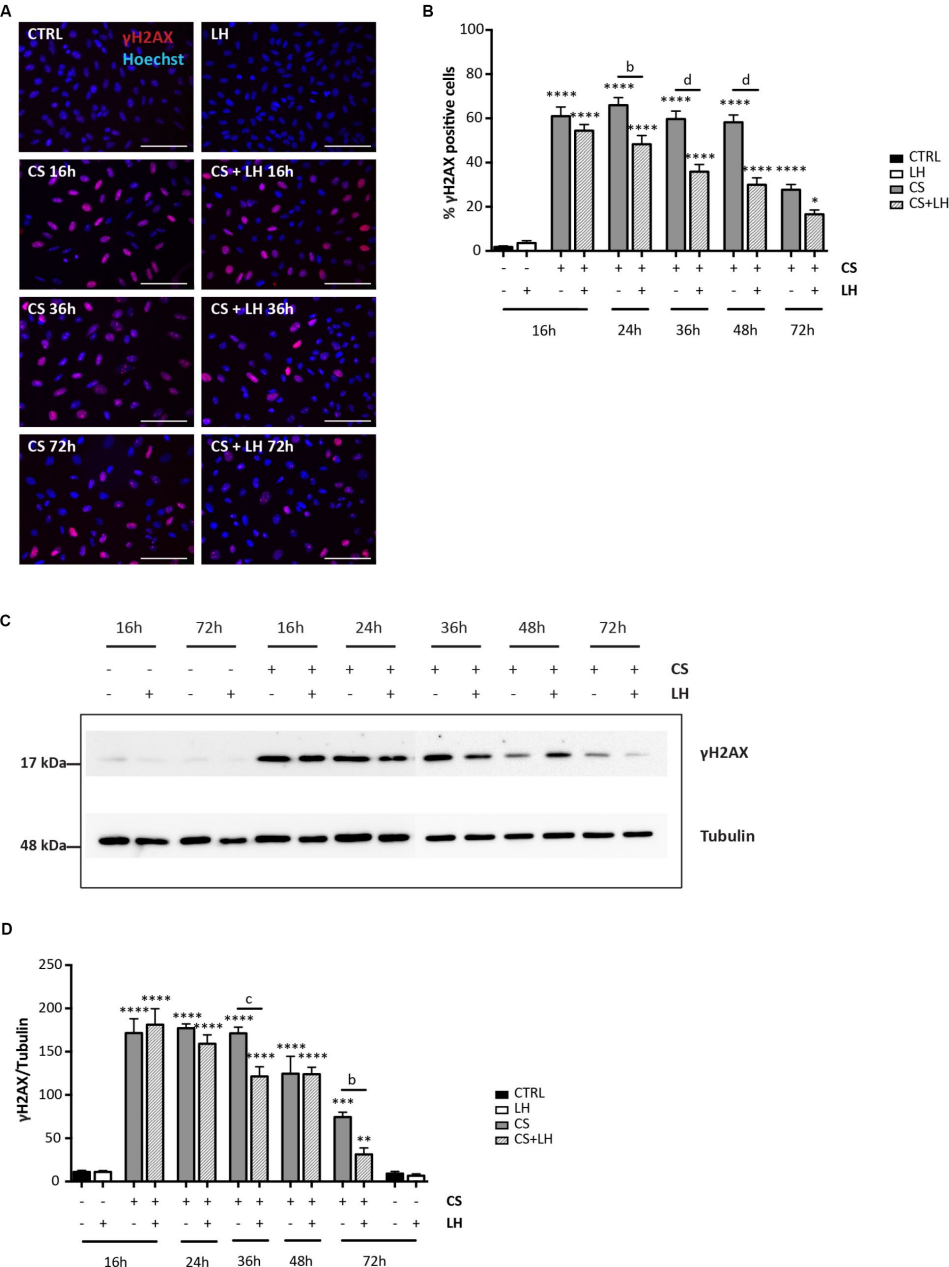
**LH modulates the H2AX phosphorylation in somatic ovarian cells.** (**A**) Representative IF for γH2AX in cultured ovarian somatic cells treated with 10 μM CS with/out 200 mIU/mL LH for the indicated times. Scale bar = 100 μm. (**B**) The graph reports the quantification of the percentage of cells positive for γH2AX. Data are shown as mean ± SEM of five experiments. Statistical differences *vs* control *p<0.05 ****p<0.0001; Cs+LH group *vs* CS group *b* = p<0.01 *d* = p<0.0001. (**C**, **D**) Representative WB of γH2AX in cells treated with CS with/out LH for the indicated times. Note a clear reduction of γH2AX level at 72 hrs. Data are expressed as mean ± SEM of three experiments. Statistical differences *vs* control **p<0.01 ***p<0.001 ****p<0.0001; statistical difference of CS group *vs* CS+LH group *b* = p<0.01 *c* = p<0.001.

**Figure 9:**
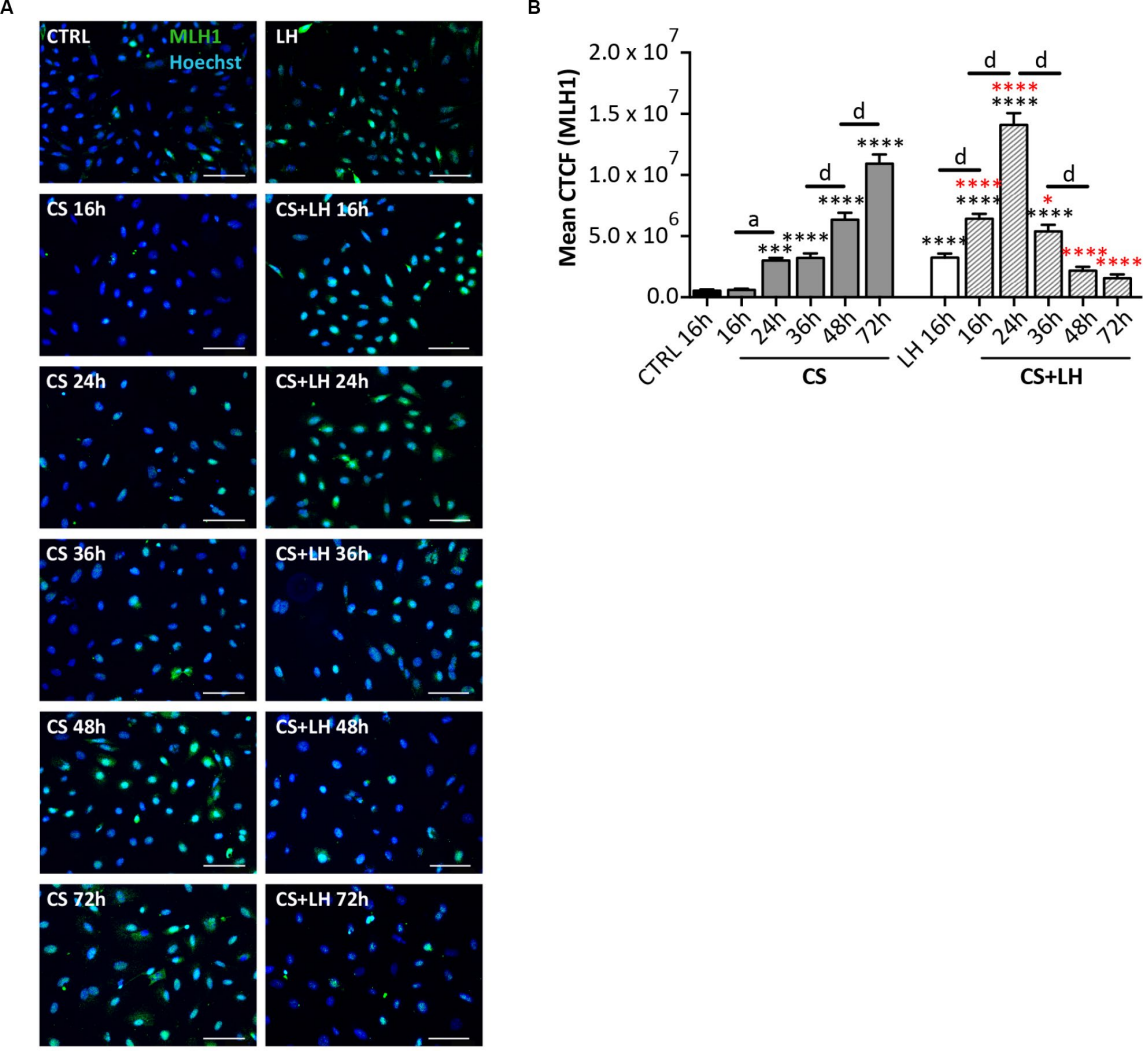
**LH improves the DNA damage repair capability by somatic ovarian cells**. (**A**) Representative IF of cells stained with MLH1 (green) after 16, 24, 36, 48 and 72 hrs of treatment with 10 μM CS with/out 200 mIU/mL LH. Scale bar = 100 μm. (**B**) The graph reports the Mean Correlated Total Cell Fluorescence (CTCF) in ovarian cells treated with/out CS and CS+LH as indicated. The fluorescence intensity was determined in each cell by ImageJ software. Data are expressed as means ± SEM of three experiments. Statistical differences *vs* control ***p<0.001 ****p<0.0001; statistical difference of CS group *vs* CS+LH group *p<0.05 ****p<0.0001; CS 16 hrs *vs* CS 24 hrs *a* p<0.05; CS 36 hrs *vs* CS 48 hrs *d* p<0.0001; CS 48 hrs *vs* CS 72 hrs *d* p<0.0001; LH 16 hrs *vs* CS+LH 16 hrs *d* p<0.0001; CS 16 hrs *vs* CS 24 hrs *d* p<0.0001; CS 24 hrs *vs* CS 36 hrs *d* p<0.0001; CS 36 hrs *vs* CS 48 hrs *d* p<0.0001. Note that cells showed a progressive increase in the nuclear expression of MLH1 already after 16 hrs of treatment with Cs+LH.

The results in Supplementary Figures 6 and 7 shown that LH was unable to counteract the DNA damages induced by EPI (Supplementary Figure 6A–6D) and the occurrence of all stress-induced processes caused by CS in the ovarian somatic cells (Supplementary Figure 7A–7H). Moreover, LH did not have a detectable effect on the initial DNA damage caused by CS measurable by γH2AX staining. However, the hormone appeared to significantly accelerate the kinetics of DNA repair occurring in CS-treated cells evaluated both by γH2AX (Figure 8A–8D) and MLH1 (Figure 9A, 9B) staining whereas it did not impinge upon the RAD51 and ERCC1 staining (Supplementary Figure 8A, 8B).

## DISCUSSION

The morphological and molecular characterization of the somatic cells from the murine prepuberal ovaries performed in the present paper, indicated the presence, in the *in vitro* culture, of cell types belonging to two major lineages, namely granulosa and stromal-theca cells. Within the granulosa cell lineage, it is possible to speculate that the scattered fibroblastoid and epithelioid FOXL2 positive cells represented phenotypes of granulosa cell precursors (pGCs) whereas the colonies of compact epithelioid cells, also positive for FOXL2, were granulosa cells (GCs) from primary and/or early secondary follicles present in small number in 8dpp ovaries. αSMA positive pre-thecal cells (pTCs) likely represent heterogeneous populations probably comprising cells expressing LHGCR. Finally, the pan-CK positive colonies of little epithelioid cells probably derived from OSE cells. Since all of these cell types in the ovary may be potential targets of chemotherapeutic agents, our *in vitro* model allowed us to investigate the individual effects of some of the most common of these drugs, such as EPI, CS and PM, on the various somatic cell populations present in prepuberal ovaries.

Considering the effect of the three tested drugs on these cell populations under the conditions used in the present work, it was evident that whereas all cell types underwent apoptosis following EPI exposure, CS and PM caused similar responses but distinct from EPI and cell type dependent. In line with our results, Morgan et al. [20] showed that GCs of primordial and growing follicles of neonatal mouse ovaries were relatively resistant *in vitro* to CS in comparison to EPI that, on the contrary, caused massive degeneration of follicular cells. The fact that these drugs act by different and cell type-dependent manner should not be surprising because their action mechanisms are discrete. In fact, EPI acts by blocking topoisomerase II activity and by intercalating into the flat space between the bases of the DNA double helix, where it can act further to disrupt DNA replication and transcription. The major mechanism of action of CS and PM is to covalently bind to DNA forming adducts (for references, see [26–29]).

In our model, the most part of the putative pTCs and pGCs did not undergo apoptosis following the addition of CS or PM to the culture medium but acquired characteristics typical of stress induced premature senescence (SIPS), a process that, as far as we know, was not previously described in such ovarian cells. Under our *in vitro* conditions, these cells undergo extensive DNA damage (γH2AX staining) and growth arrest (likely in G2/M), testified by flow cytometric analyses, in situ proliferation assay, decrease of *Ki67* and increase of *p21* transcripts, within 24 hrs of drug exposure. Moreover, they acquired a SIPS phenotype (large and flat morphology and SA-βgal activity) from 48-72 hrs onwards. On the other hand, the putative GCs did not undergo apoptosis and did not acquire the morphological SIPS features. Since SIPS occurred primarily in proliferating cells, the fact that at the time of the drug addiction these cells were, according to the Click-iT EdU assay, already cell cycle arrested because confluence is the likely explanation. A possibility supported by the fact that they were positive for SA-βgal activity also in the control condition [23]. Moreover, both putative GCs from 8dpp ovaries and GCs from secondary follicles from 16dpp ovaries, appeared resistant to DNA damage caused by CS and evaluated by γH2AX staining. Many studies made in cell lines demonstrated that CS resistance might be mediated through two broad mechanisms: first, a failure of enough drug to reach the target DNA, and second, a failure to achieve cell death after CS–DNA adduct formation. Many resistant cells show a pleomorphic phenotype consisting of altered pathways involving drug uptake, DNA-damage recognition and repair, and apoptosis (for a review, see [30]). Which mechanisms make GCs apparently resistant to CS remain to be investigated. Conversely, putative OSE cells were also resistant to CS-induced apoptosis and SIPS but not to DNA damage. Moreover, the majority of them, whilst repairing DNA damage after 72 hrs of drug treatment, continued to proliferate.

Regarding SIPS, here observed induced by CS and PM in pTCs and pGCs, it is generally thought that it is a form of cell death, irreversibly eliminating replicating cells. What directs a cell to undergo senescence or apoptosis remains unclear, but cell type, the kind of damaging agent and the dose administered may be important [31].

In our culture system, cells underwent SIPS following G2/M arrest and were characterized by increased level of p21 expression rather than p16 (this latter not shown). Moreover, in contrast to the notion reported above, in about half of SIPS-cells, γH2AX foci were resolved after 72 hrs of drug treatment. The marked increase of the MLH1 staining in the most part of cells preceding the decrease of the γH2AX foci, confirmed the kinetics of the DNA repair and suggested the involvement of the MMR in such process. Evidence of other DNA repair pathways such as HR (RAD51 staining) and NER (ERRC1 staining) was not found.

Although, these findings suggest that DNA damage was recovered by such cells, it remained to be determined if all DNA damages were actually repaired. As a matter of fact, these cells maintained the SIPS phenotype and growth arrest at least for the following 7 days of culture (not shown).

The observation that CS and PM caused expression of cell cycle arrest markers also in cultured ovarian fragments (increased p21 and ki67 transcripts) and in ovaries *in vivo* (increased p21 transcripts) of comparable age, make it unlikely that SIPS is artificially induced by the drug as a consequence of the ovarian tissue disaggregation and/or to the *in vitro* conditions. This conclusion opens the question of the possible impact that such process has on the ovary under various stress agents including chemotherapy or during aging. In fact, whereas there is little doubt that SIPS *in vivo* can occur in a variety of tissues [32], less clear are its dynamics and consequences. Evidence exists that SIPS can suppress proliferation in cancer cells; however, senescent cells may be prone to genetic and epigenetic instability, which is also a hallmark of cancer cells [33]. Senescent cells can occur transiently (e.g., during embryogenesis, wound healing and tissue injury), with beneficial effects on tissue homeostasis and regeneration, or accumulate chronically in tissues detrimentally affecting the microenvironment by de- or trans- differentiation of senescent cells and their neighboring stromal cells [34]. A part of these latter effects is attributable to the fact that SIPS-cells are metabolically active and have undergone widespread changes in protein expression and secretion, ultimately developing the senescence-associated secretory phenotype (SASP) [33, 35]. SASP includes the secretion of numerous pro-inflammatory cytokines, chemokines, growth factors, and proteases that can have powerful either beneficial or deleterious paracrine activities depending on the physiological environment [36, 37]. In such a context, although the present data show that LH did not significantly influence the occurrence of SIPS, it remains possible that the hormone exerts an important control on SASP and on the anti-apoptotic action that the hormone responsive cells have on the primordial follicle reserve in the prepuberal and adult ovary [17]. On the other hand, LH appears to have an important positive action on the MMR pathways activated in the ovarian cells following the DNA damage caused by CS. The way through which the hormone regulates such process and its possible influence on SASP are object of ongoing researches.

## MATERIALS AND METHODS

### Animals

CD-1 and transgenic p18 GFP/c-Kit [38] mice were housed and mated under standard laboratory conditions in an environmentally controlled room and treated using humane care in order to inflict the least possible pain. All experiments were approved by the Institutional Animal Care and Use Committee (IACUC) and carried out according to the Italian and European rules (D.L.116/92; C.E. 609/86; European Directive 2010/63/EU); authorization n°391/2016-PR.

### Compounds and antibodies

Epirubicin (EPI) and Cisplatin (CS) were purchased from Sigma-Aldrich and dissolved in water and DMSO, respectively. Phosphoramide mustard (PM), the active metabolite of Cyclophosphamide, from National Cancer Institute and dissolved in DMSO. Human recombinant LH was a gift from Merck KGaA (Germany).

The following primary antibodies were used: Tubulin (T9026, Sigma-Aldrich); GADPH (G9545, Sigma-Aldrich); LHCGR (sc-25828, Santa Cruz); FOXL2 (ab5096, Abcam); γH2AX (05-636, Millipore); pan-cytokeratin (pan-CK, Z0622, Dako); αSMA (A2547, Sigma-Aldrich); RAD51 (sc-8349, Santa Cruz Biotechnology); ERCC1 (sc-17809, Santa Cruz Biotechnology); MLH1 (sc-271978, Santa Cruz Biotechnology). HRP-conjugated secondary antibodies were as follows: donkey anti-goat (Jackson ImmunoResearch) (1:50000); goat anti-mouse (GE Healthcare) (1:5000); goat anti-rabbit (GE Healthcare) (1:5000). The following secondary antibodies were used for IF analysis: Cy3-labeled donkey anti-goat antibody (1:400); Alexa Fluor 488-labeled goat anti-mouse (Thermo Fisher Scientific) (1:500); Alexa Fluor 568-labeled goat anti-mouse (Thermo Fisher Scientific) (1:500); Alexa Fluor 488-labeled goat anti-rabbit (Thermo Fisher Scientific) (1:500).

### *In vivo* treatment of animals

For each experimental group, twelve 8 *days post-partum* (dpp) CD1 mice were used. They were intraperitoneally injected with 30 μl of physiological solution or equal volume of CS (5 mg/Kg).

### *In vitro* culture of ovarian somatic cells

Ovaries were collected from 8dpp CD1 mice, cleared of the surrounding tissues and incubated in 1 mg/mL collagenase type I (Sigma-Aldrich) in αMEM for 1 hr at 37°C in agitation. Following collagenase removal, the ovaries were suspended in the cultured medium (see below) and disaggregated by gently pipetting. Cell suspension was filtered through a 40 μm nylon filter (BD Falcon) while the retained tissue fragments were further incubated in 0.25% trypsin (Sigma-Aldrich) for 15 min at 37°C in agitation, disaggregated and filtered as above. The cellular suspensions were then mixed and finally plated at 30.000 cells/cm^2^. Culture was carried out in αMEM (Aurogene) supplemented with 10% FBS (Gibco), L-glutamine, penicillin-G and streptomycin, pyruvic acid, N-acetyl-L-cysteine (all from Sigma-Aldrich) and ITS liquid media supplement (Gibco), at 37°C in 5% CO_2_ in 95% humidified incubator. The day after ( = T0 time point), 0.5 μM EPI, 10 μM CS or 10 μM PM was added to the culture medium of the treated groups. Where indicated, 200 mIU/mL LH was also added to the medium 1 hr before drugs.

Images were acquired under phase contrast microscope (Leitz Diavert) equipped with a DS-Fi1 camera (Nikon).

### Culture of ovarian fragments

Ovaries were collected from 4dpp transgenic p-18 GFP/c-kit mice as reported in Rossi, Lispi [17]. Briefly, each ovary was cleaned and fragmented in 8 pieces. Fragments were, then, plated at 37°C and 5% CO_2_ in αMEM (Aurogene) supplemented with 10% FBS (Gibco), L-glutamine, penicillin-G and streptomycin, pyruvic acid, N-acetyl-L-cysteine and ITS liquid media supplement (all from Sigma-Aldrich). After 4 days in culture, the ovarian fragments formed a thin layer of tissue containing GFP-positive oocyte visible under a fluorescence microscope. At this time (T0 time point), drugs were added to the culture medium at the concentrations indicated above. For the co-treatments with LH and chemotherapeutic agent, cells were pre-incubated with LH 1 hr before the addition of the drug. The number of morphologically healthy GFP-positive oocytes with a diameter of 20 μm (POs) was scored at T0 and after 24 hrs of treatment under a Leica CTR 6000 microscope (Watzlar, Germany).

The values presented are the mean ± SEM of at least three independent experiments in which at least three fragments were scored for each experimental condition at T0 and after 24 hrs of culture.

### Culture of follicular cells from secondary follicles

Ovaries were dissected from 16dpp CD1 mice, cleaned from the adipose tissues and placed in αMEM supplemented with 1mg/ml BSA (Sigma-Aldrich) and 25 mM HEPES (Gibco). They were punctured and minced in order to release follicles and those with more than one granulosa cells layer (early secondary follicles) were washed and transferred in culture medium using a glass micropipette to be cultured at 37°C in 5% CO_2_ in 95% humidified incubator. Within 24 hrs, the follicle cells spread usually releasing the oocyte in the medium to form a compact epithelioid cell monolayer.

### Flow cytometry

Adherent cells were collected and fixed in cold methanol/acetone (1:4) O.N. at 4°C. After incubation with RNase (100 μg/mL in PBS) for 20 min at room temperature, cellular suspension was marked with propidium iodide (1 mg/mL in PBS, from Sigma-Aldrich) for 20 min at room temperature. Samples were analyzed on a FACSCalibur (BD Biosciences) with Cell Quest Pro software (Becton Dickinson). In each sample, 10000 events were counted. Data analysis was conducted with FlowJo software (Tree Star).

### Immunohistochemistry and immunofluorescence

For immunohistochemistry (IHC) and immunofluorescence (IF) on cultured cells, medium was removed and, after two-three washes in PBS, cells were fixed with 4% paraformaldehyde (PFA) for 15 min at room temperature. For IF analysis on ovarian sections, ovaries were collected and fixed in 4% PFA for 4h. After dehydration, tissues were embedded in paraffin and sectioned (5 μm sections) following standard procedures. After deparaffinization and hydration, antigen retrieval in citrate buffer (pH 6) was performed. Both cultured cells and ovarian sections were permeabilized with 0.1% Triton X in PBS for 10 min, blocked in 3% BSA for 30 min and then incubated with primary antibodies O.N. at 4°C: FOXL2 (1:200); αSMA (1:300); pan-CK (1:250); γH2AX (1:500); RAD51 (1:100); MLH1 (1:100); ERCC1 (1:100). Primary antibody binding was detected using fluorescent-dye conjugated secondary antibody (1 hr at room temperature). Hoechst was used as counterstain. Both cells and sections were mounted in PBS-glycerol (1:1) and analyzed by Leica DMI6000B microscope. For quantification of different cell populations in culture, at least 150 cells within randomly selected field were scored for a total of 500 cells in each separate experiment. For γH2AX staining, cells were considered positive if they displayed >4 nuclear dots or a fully stained nucleus [39, 40]; for each condition, at least 300 cells were scored in each separate experiment. For fluorescence intensity analysis, at least 300 cells were analyzed in each separate experiment using ImageJ software. The Correlated Total Cell Fluorescence (CTCF) for each cell was calculated using the following equation: CTCF = Integrated Density of selected cell – (Area of selected cell * Mean fluorescence of background readings). Data obtained were represented in figure as average of all cells analyzed.

### Click-iT Plus EdU and SA-βgal staining

The ability of cells to proliferate was estimated using Click-iT Plus EdU (Cat. n. C10637) purchased from Molecular Probe according to the manufacturer’s instructions.

SA-βgal activity was detected as reported by Debacq-Chainiaux et al. [23]. Briefly, cells were fixed (2% formaldehyde/0,2% glutaraldehyde), and incubated with a staining solution, (40 mM citric acid/Na phosphate buffer, 5 mM K4[Fe(CN)6] 3H_2_O, 5 mM K3[Fe(CN)6], 150 mM sodium chloride, 2 mM magnesium chloride and 1 mg/mL X-gal in distilled water). Staining was analyzed under a phase contrast microscope.

### Total RNA extraction and cDNA synthesis

Briefly, RNA was extracted from cultured cells, tissue fragments or ovaries using TRIzol (Invitrogen) following the manufacturer’ instructions; RNA was then quantified with NanoDrop ND-1000 Spectrophotometer (Thermo Fisher Scientific). The first strand cDNA was synthesized from 500 μg of RNA template by *TransScript One-Step gDNA Removal and cDNA Synthesis SuperMix* kit (TransGene Biotech) in a 20 μl reaction.

### RT-PCR amplification

For PCR amplification, 50 ng of cDNA were used. PCR was performed using 0.5 μM of forward and reverse primers (obtained from Sigma-Aldrich) and *PCR Master Mix kit* (Thermo Fiser Scientific) in a total volume of 20 μl. Amplifications were performed using a PCR Thermal cycler (Mastercycler, Eppendorf) as follows: 1) initial denaturation at 95°C for 3 min, 2) followed by 35 cycles of denaturation at 95°C for 30 sec, primer annealing at 55°C (for GAPDH, AMH and LHCGR) or 60°C (for FOXL2, Gli1 and CYP17A1) for 1 min and primer extension at 72°C for 1 min, 3) finally, 7 min at 72°C to ensure extension of remaining single strands. The reaction products were analyzed by electrophoresis on 1,5% agarose gels and visualized by ethidium bromide staining under an UV light. GAPDH was used as internal control. GAPDH fw: AACTTTGG CATTGTGGAAGG, GAPDH rv: CCGTGTTCCTAC CCCCAATGTG; LHCGR fw: CCTGAGCATCTGTA ACACAG, LHCGR rv: TTCCTGAAAGCACAGCA GTG; Gli1 fw: TCCACAGGCATACAGGATCA, Gli1 rv: TGCAACCTTCTTGCTCACAC; CYP17A1 fw: TC TGGGCACTGCATCACG, CYP17A1 rv: GCTCCGA AGGGCAAATAACT; AMH fw: GCAGTTGCTAGTC CTACATC, AMH rv: TCATCCGCGTGAAACAGCG; FOXL2 fw: AAGCCCCCGTACTCGTACGTGGCGC TCATC, FOXL2 rv: GTAGTTGCCCTTCTCGAACA TGTC.

### Real-time PCR (qRT-PCR)

30 ng of reverse-transcribed cDNA was used for Real Time polymerase chain reaction (PCR) using iTaq Universal SYBR Green Supermix (Biorad) and specific forward (F) and reverse (R) primers (from Sigma-Aldrich). Primers were used at a concentration of 0.5 μM and the reaction performed on a 7300 Real-Time PCR System (Applied Biosystems). The thermal cycling conditions were Data from the reaction were collected and analyzed using the 2^-ΔΔCt^ method. Relative quantization of gene expression was performed relating the signal in the treated group to that of the untreated group. GAPDH was used as internal reference. GAPDH fw: AACTTTGGCATTGTGGAAGG, GAPDH rv: CCGTGTTCCTACCCCCAATGTG; P21 fw: GCAGA TCCACAGCGATATCC, P21 rv: CAACTGCT CACTGTCCACGG; Ki-67 fw: AATCCAACT CAAGTAAACGGGG, Ki-67 rv: TTGGCTTGCTT CCATCCTCA.

### Western blotting

For Western Blotting (WB) analysis, samples were lysed in a solution containing 50 mM Tris-HCl pH 7.4, 150 mM NaCl, 0.5% NP-40, 5 mM EDTA, 0.5% sodium deoxycholate, 1 mM PMSF, 1mM sodium o-vanadate, and protease inhibitors (all from Sigma-Aldrich). Samples were homogenized on ice by ultrasonic homogenization and proteins concentration determined by Bradford assay.

Proteins (about 30 μg) were resolved on SDS-polyacrylamide gel and transferred to a PVDF transfer membrane (GE Healthcare). Blots were blocked in 5% non-fat dry milk in PBS-T (0.05% Tween 20 in PBS) for 1h at room temperature and then incubated with primary antibodies (in 1% milk in PBS-T) O.N. at 4°C: Tubulin (1:1000); GAPDH (1:2000); LHR (1:200); FOXL2 (1:1000); γH_2_AX (1:1000). Membranes were, then, incubated with HRP-conjugated secondary antibody for 1 h at room temperature and signals detected by peroxidase reaction using *Clarity Western ECL Substrate* (Biorad). Immunoblots were quantitatively evaluated using ImageJ software (NIH).

### Statistical analysis

Data were analyzed with GraphPad Prism (software version 7.0, San Diego, CA). Results were given as mean ± SEM and *P* value was determined by one-way Anova and Bonferroni post-analyses. Statistical significance was based on *P* value: *p< 0.05, **p<0.01, ***p< 0.001, ****p<0.0001; *a* = p< 0.05, *b* = p<0.01, *c* = p< 0.001, *d* = p<0.0001.

## Supplementary Material

Supplementary Figures

## References

[1] Coulam CB, Adamson SC, Annegers JF. Incidence of premature ovarian failure. Obstet Gynecol. 1986; 67:604–06. 3960433

[2] Pederson J, Kumar RB, Adams Hillard PJ, Bachrach LK. Primary ovarian insufficiency in adolescents: a case series. International Journal of Pediatric Endocrinology. 2015; 2015:13. 10.1186/s13633-015-0009-z 25983758 PMC4433018

[3] De Felici M. Embriologia umana. Morfogenesi, processi molecolari, aspetti clinici: Piccin-Nuova Libraria 2013.

[4] Gougeon A. Dynamics of Human Follicular Growth: Morphologic, Dynamic, and Functional Aspects. The ovary, second edition. 2004 10.1016/b978-012444562-8/50003-3

[5] Hsueh AJ, Billig H, Tsafriri A. Ovarian follicle atresia: a hormonally controlled apoptotic process. Endocr Rev. 1994; 15:707–24. 10.1210/edrv-15-6-707 7705278

[6] Marcozzi S, Rossi V, Salustri A, De Felici M, Klinger FG. Programmed cell death in the human ovary. Minerva Ginecol. 2018; 70:549–60. 10.23736/S0026-4784.18.04274-0 29999289

[7] Wallace WH, Kelsey TW. Human ovarian reserve from conception to the menopause. PLoS One. 2010; 5:e8772. 10.1371/journal.pone.0008772 20111701 PMC2811725

[8] Hansen KR, Knowlton NS, Thyer AC, Charleston JS, Soules MR, Klein NA. A new model of reproductive aging: the decline in ovarian non-growing follicle number from birth to menopause. Hum Reprod. 2008; 23:699–708. 10.1093/humrep/dem408 18192670

[9] Webber L, Davies M, Anderson R, Bartlett J, Braat D, Cartwright B, Cifkova R, de Muinck Keizer-Schrama S, Hogervorst E, Janse F, Liao L, Vlaisavljevic V, Zillikens C, Vermeulen N, and European Society for Human Reproduction and Embryology (ESHRE) Guideline Group on POI. ESHRE Guideline: management of women with premature ovarian insufficiency. Hum Reprod. 2016; 31:926–37. 10.1093/humrep/dew027 27008889

[10] American Cancer Society, Merck KGaA. Global burden of cancer in women. Current status, trends, and interventions. 2016.

[11] van Dorp W, Haupt R, Anderson RA, Mulder RL, van den Heuvel-Eibrink MM, van Dulmen-den Broeder E, Su HI, Winther JF, Hudson MM, Levine JM, Wallace WH. Reproductive Function and Outcomes in Female Survivors of Childhood, Adolescent, and Young Adult Cancer: A Review. J Clin Oncol. 2018; 36:2169–80. 10.1200/JCO.2017.76.3441 29874135 PMC7098836

[12] Recchia F, Saggio G, Amiconi G, Di Blasio A, Cesta A, Candeloro G, Rea S. Gonadotropin-releasing hormone analogues added to adjuvant chemotherapy protect ovarian function and improve clinical outcomes in young women with early breast carcinoma. Cancer. 2006; 106:514–23. 10.1002/cncr.21646 16388519

[13] Arrigo T, Bertelloni S, Carcione L, De Luca F, De Sanctis C, Einaudi S, Pirazzoli P, Segni M, Urso L, Wasniewska M. Characterization of early presentation idiopathic ovarian failure in girls and adolescents. J Pediatr Endocrinol Metab. 2003; 16:835–42. 10.1515/JPEM.2003.16.6.835 12948295

[14] Papagianni V, Deligeoroglou E, Makrakis E, Botsis D, Creatsas G. Response to hormonal treatment of young females with primary or very premature ovarian failure. Gynecol Endocrinol. 2011; 27:291–99. 10.3109/09513591003632274 21381875

[15] Kim SY, Kim SK, Lee JR, Woodruff TK. Toward precision medicine for preserving fertility in cancer patients: existing and emerging fertility preservation options for women. J Gynecol Oncol. 2016; 27:e22. 10.3802/jgo.2016.27.e22 26768785 PMC4717227

[16] Spears N, Lopes F, Stefansdottir A, Rossi V, De Felici M, Anderson RA, Klinger FG. Ovarian damage from chemotherapy and current approaches to its protection. Hum Reprod Update. 2019. [Epub ahead of print]. 10.1093/humupd/dmz027 31600388 PMC6847836

[17] Rossi V, Lispi M, Longobardi S, Mattei M, Di Rella F, Salustri A, De Felici M, Klinger FG. LH prevents cisplatin-induced apoptosis in oocytes and preserves female fertility in mouse. Cell Death Differ. 2017; 24:72–82. 10.1038/cdd.2016.97 27689876 PMC5260508

[18] Mork L, Maatouk DM, McMahon JA, Guo JJ, Zhang P, McMahon AP, Capel B. Temporal differences in granulosa cell specification in the ovary reflect distinct follicle fates in mice. Biol Reprod. 2012; 86:37. 10.1095/biolreprod.111.095208 21976597 PMC3290667

[19] Liu C, Peng J, Matzuk MM, Yao HH. Lineage specification of ovarian theca cells requires multicellular interactions via oocyte and granulosa cells. Nat Commun. 2015; 6:6934. 10.1038/ncomms7934 25917826 PMC4413950

[20] Morgan S, Lopes F, Gourley C, Anderson RA, Spears N. Cisplatin and doxorubicin induce distinct mechanisms of ovarian follicle loss; imatinib provides selective protection only against cisplatin. PLoS One. 2013; 8:e70117. 10.1371/journal.pone.0070117 23922929 PMC3726485

[21] Pfeifle CE, Howell SB, Felthouse RD, Woliver TB, Andrews PA, Markman M, Murphy MP. High-dose cisplatin with sodium thiosulfate protection. J Clin Oncol. 1985; 3:237–44. 10.1200/JCO.1985.3.2.237 4038510

[22] Tropitzsch A, Arnold H, Bassiouni M, Müller A, Eckhard A, Müller M, Löwenheim H. Assessing cisplatin-induced ototoxicity and otoprotection in whole organ culture of the mouse inner ear in simulated microgravity. Toxicol Lett. 2014; 227:203–12. 10.1016/j.toxlet.2014.03.022 24709139

[23] Debacq-Chainiaux F, Erusalimsky JD, Campisi J, Toussaint O. Protocols to detect senescence-associated beta-galactosidase (SA-betagal) activity, a biomarker of senescent cells in culture and in vivo. Nat Protoc. 2009; 4:1798–806. 10.1038/nprot.2009.191 20010931

[24] Desmeules P, Devine PJ. Characterizing the ovotoxicity of cyclophosphamide metabolites on cultured mouse ovaries. Toxicol Sci. 2006; 90:500–9. 10.1093/toxsci/kfj086 16381661

[25] Jardine I, Fenselau C, Appler M, Kan MN, Brundrett RB, Colvin M. Quantitation by gas chromatography-chemical ionization mass spectrometry of cyclophosphamide, phosphoramide mustard, and nornitrogen mustard in the plasma and urine of patients receiving cyclophosphamide therapy. Cancer Res. 1978; 38:408–15. 10.1007/978-1-349-03328-7_8 620410

[26] Gómez-Ruiz S, Maksimović-Ivanić D, Mijatović S, Kaluđerović GN. On the discovery, biological effects, and use of Cisplatin and metallocenes in anticancer chemotherapy. Bioinorg Chem Appl. 2012; 2012:140284. 10.1155/2012/140284 22844263 PMC3401524

[27] Siddik ZH. Cisplatin: mode of cytotoxic action and molecular basis of resistance. Oncogene. 2003; 22:7265–79. 10.1038/sj.onc.1206933 14576837

[28] McGowan JV, Chung R, Maulik A, Piotrowska I, Walker JM, Yellon DM. Anthracycline Chemotherapy and Cardiotoxicity. Cardiovasc Drugs Ther. 2017; 31:63–75. 10.1007/s10557-016-6711-0 28185035 PMC5346598

[29] Thorn CF, Oshiro C, Marsh S, Hernandez-Boussard T, McLeod H, Klein TE, Altman RB. Doxorubicin pathways: pharmacodynamics and adverse effects. Pharmacogenet Genomics. 2011; 21:440–46. 10.1097/FPC.0b013e32833ffb56 21048526 PMC3116111

[30] Kelland L. The resurgence of platinum-based cancer chemotherapy. Nat Rev Cancer. 2007; 7:573–84. 10.1038/nrc2167 17625587

[31] Lee JJ, Kim BC, Park MJ, Lee YS, Kim YN, Lee BL, Lee JS. PTEN status switches cell fate between premature senescence and apoptosis in glioma exposed to ionizing radiation. Cell Death Differ. 2011; 18:666–77. 10.1038/cdd.2010.139 21072054 PMC3131905

[32] Lee S, Schmitt CA. The dynamic nature of senescence in cancer. Nat Cell Biol. 2019; 21:94–101. 10.1038/s41556-018-0249-2 30602768

[33] Schosserer M, Grillari J, Breitenbach M. The Dual Role of Cellular Senescence in Developing Tumors and Their Response to Cancer Therapy. Front Oncol. 2017; 7:278. 10.3389/fonc.2017.00278 29218300 PMC5703792

[34] Banito A, Lowe SW. A new development in senescence. Cell. 2013; 155:977–78. 10.1016/j.cell.2013.10.050 24267881 PMC4702512

[35] Kuilman T, Peeper DS. Senescence-messaging secretome: SMS-ing cellular stress. Nat Rev Cancer. 2009; 9:81–94. 10.1038/nrc2560 19132009

[36] Childs BG, Baker DJ, Kirkland JL, Campisi J, van Deursen JM. Senescence and apoptosis: dueling or complementary cell fates? EMBO Rep. 2014; 15:1139–53. 10.15252/embr.201439245 25312810 PMC4253488

[37] Rodier F, Campisi J. Four faces of cellular senescence. J Cell Biol. 2011; 192:547–56. 10.1083/jcb.201009094 21321098 PMC3044123

[38] Cairns LA, Moroni E, Levantini E, Giorgetti A, Klinger FG, Ronzoni S, Tatangelo L, Tiveron C, De Felici M, Dolci S, Magli MC, Giglioni B, Ottolenghi S. Kit regulatory elements required for expression in developing hematopoietic and germ cell lineages. Blood. 2003; 102:3954–62. 10.1182/blood-2003-04-1296 12907433

[39] Clingen PH, Wu JY, Miller J, Mistry N, Chin F, Wynne P, Prise KM, Hartley JA. Histone H2AX phosphorylation as a molecular pharmacological marker for DNA interstrand crosslink cancer chemotherapy. Biochem Pharmacol. 2008; 76:19–27. 10.1016/j.bcp.2008.03.025 18508035

[40] Olive PL, Banáth JP. Kinetics of H2AX phosphorylation after exposure to cisplatin. Cytometry B Clin Cytom. 2009; 76:79–90. 10.1002/cyto.b.20450 18727058

