# Supplementary material for: Distinct effects of epirubicin, cisplatin and cyclophosphamide on ovarian somatic cells of prepuberal ovaries
Source: Aging (Albany NY). 2019 Nov 11;11(22):10532–56. doi: 10.18632/aging.102476 (PMC6914390; doi:10.18632/aging.102476)
Supplement: Supplementary Figures [file aging-11-102476-s001..pdf]

SUPPLEMENTARY FIGURES

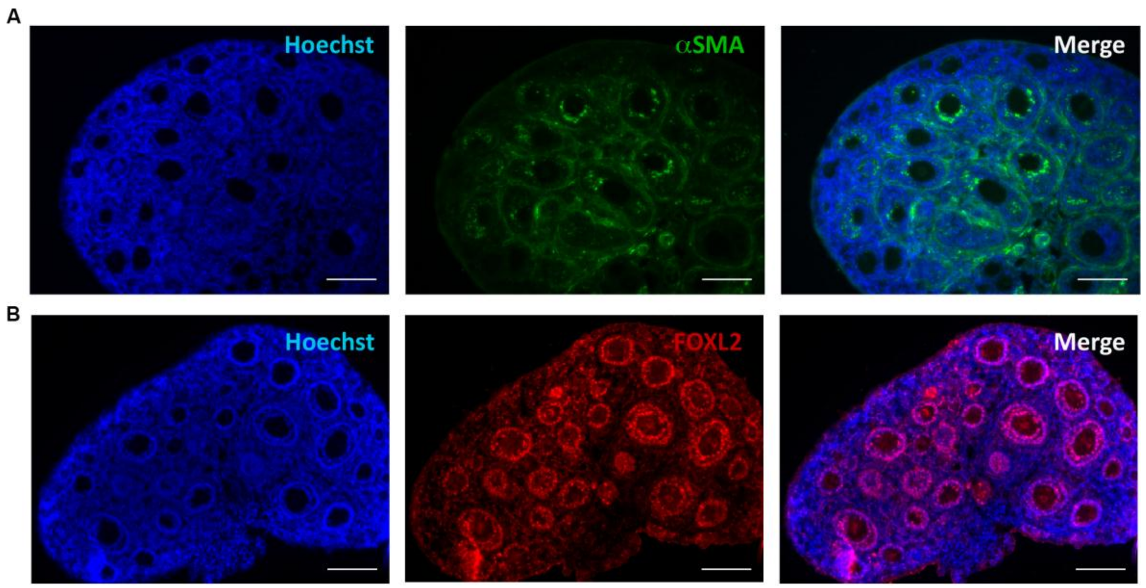

Supplementary Figure 1. IF analysis of 8dpp ovaries. Representative IF in histological sections of 8dpp ovaries showing (**A**) αSMA (green) and (**B**) FOXL2 (red) positivity in cells at the periphery and in GC layers of growing follicles, respectively. Scale bar = 100μm.

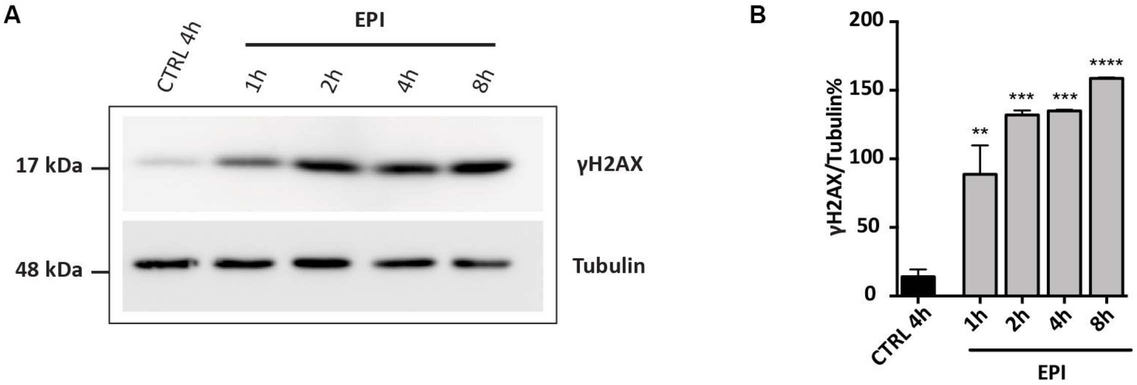

Supplementary Figure 2. WB analysis of γH2AX expression in ovarian somatic cells treated with EPI. (**A**) Representative WB for γH2AX in cultured ovarian somatic cells treated with 0.5μM EPI for the indicated times. (**B**) Densitometric quantification of the relative expression of γH2AX normalized against tubulin. Data are expressed as mean ± SEM of three analyses. Statistical differences *vs* control **p<0.01 ***p<0.001 ****p<0.0001.

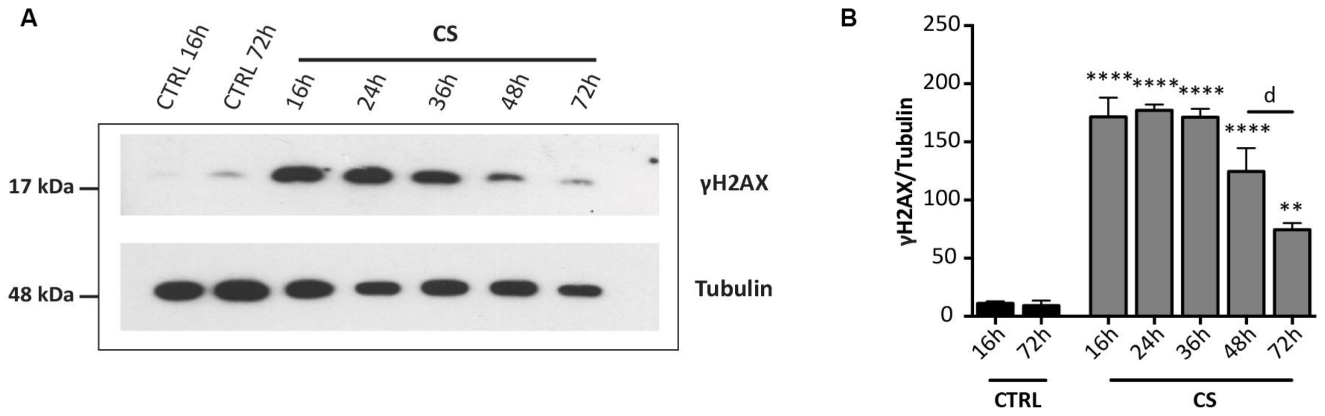

Supplementary Figure 3. Analysis of CS-induced senescence in ovarian somatic cells. (**A**) Representative WB of γH2AX expression in ovarian somatic cells treated with CS. (**B**) Densitometric quantification of γH2AX level normalized by tubulin. Data are expressed as mean± SEM of four analyses. Statistical differences *vs* control **p<0.01 ****p<0.0001; CS 48 hrs *vs* CS 72 hrs *d* = p<0.0001.

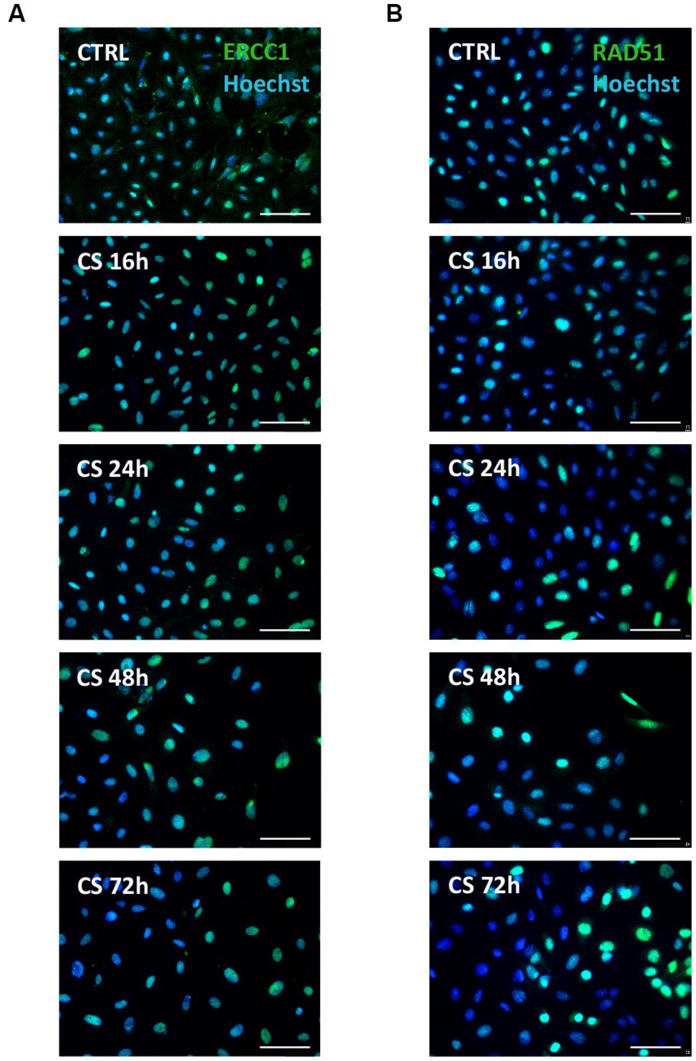

Supplementary Figure 4. Analysis of ERCC1 and RAD51 expression after CS treatment. (**A**, **B**) Representative IF of cells stained with ERCC1 (green, **A**) or RAD51 (green, **B**) after 16, 24, 48 and 72 hrs of treatment with 10 μM CS. Scale bar = 100 μm.

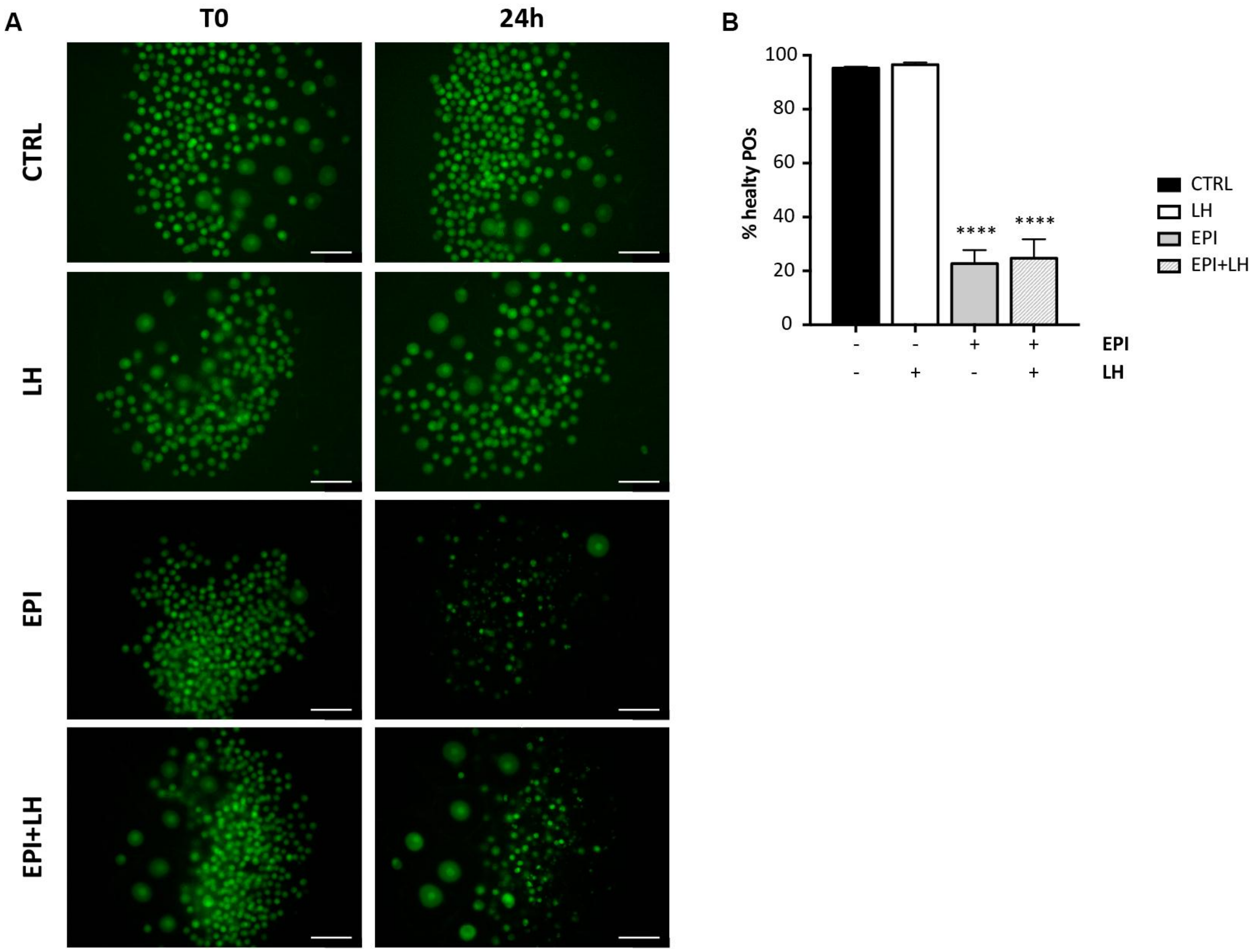

Supplementary Figure 5. LH has no effect on EPI-induced apoptosis of POs. (**A**) Ovarian fragments from P4 ovaries of p-18 c-Kit/GFP mice cultured for 4 days (T0) and incubated for 24 hrs in the presence 0.5μM EPI with/out 200mIU/mL LH, scale bar = 100μm. (**B**) Percentage of healthy GFP oocytes after 24 hrs of culture in the presence of the indicated compounds; only GFP-positive oocytes with a diameter < 20 μm (within PMF) were considered for the score (Rossi et al., 2017). Data are represented as mean ± SEM of three experiments. Statistical differences *vs* control ****p<0.0001.

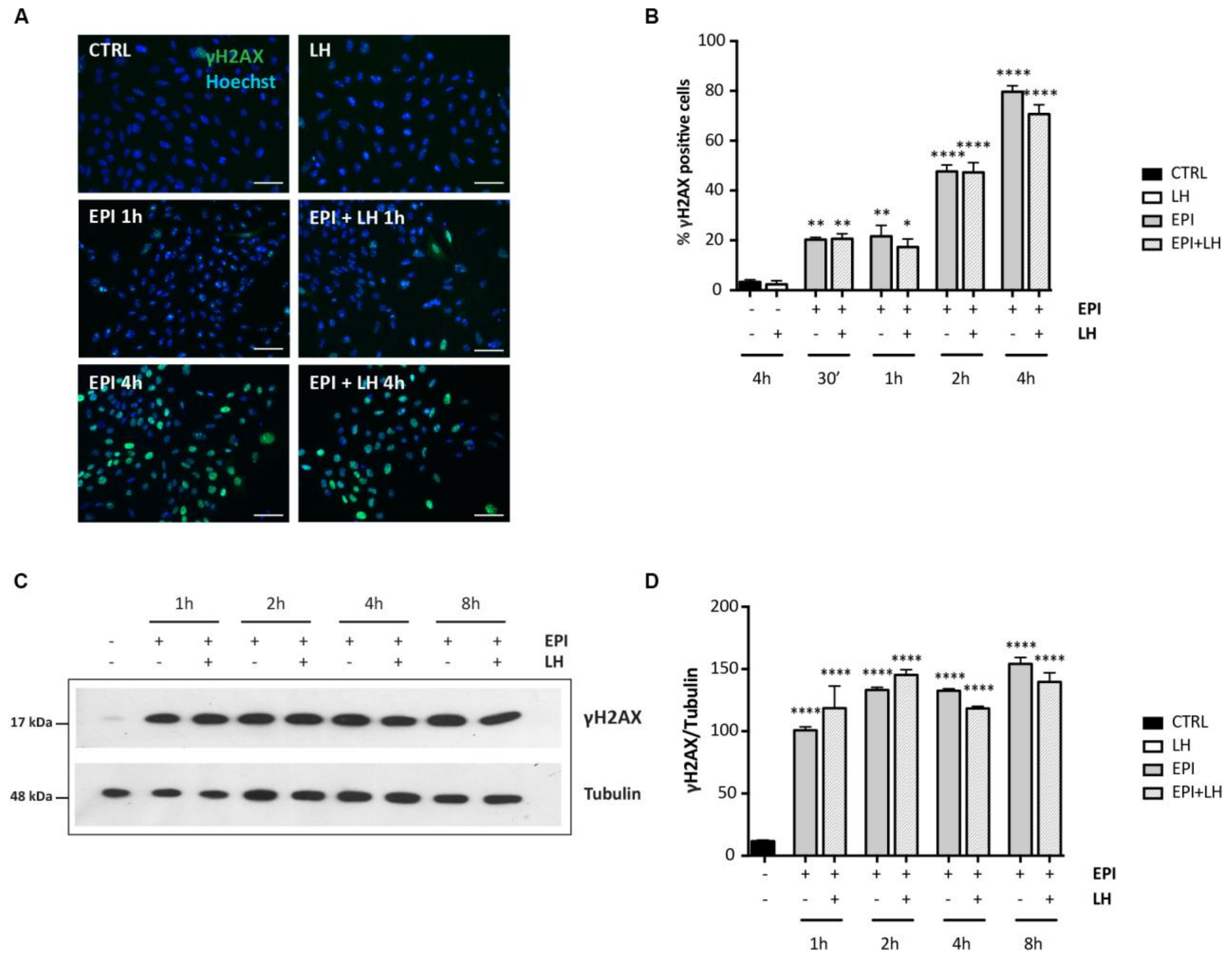

Supplementary Figure 6. LH has no effect on DNA damages induced by EPI in ovarian somatic cells. (**A**) Representative IF and quantification of ovarian somatic cells stained for γH2AX (green) after 30', 1 hr and 4 hrs of treatment with 0.5 μM EPI with/out 200 mIU/mL LH, scale bar = 100 μm. (**B**) The graph reports the quantification of the percentage of cells positive for γH2AX. Data are reported as mean ± SEM of percent of γH2AX positive cells scored in three experiments. Statistical differences *vs* control *p<0.05 **p<0.01 ****p<0.0001. (**C**) Representative WB and (**D**) densitometric quantification of γH2AX in the same groups up to 8 hrs. Data are shown as mean ± SEM of three analyses. Statistical differences *vs* control ****p<0.0001.

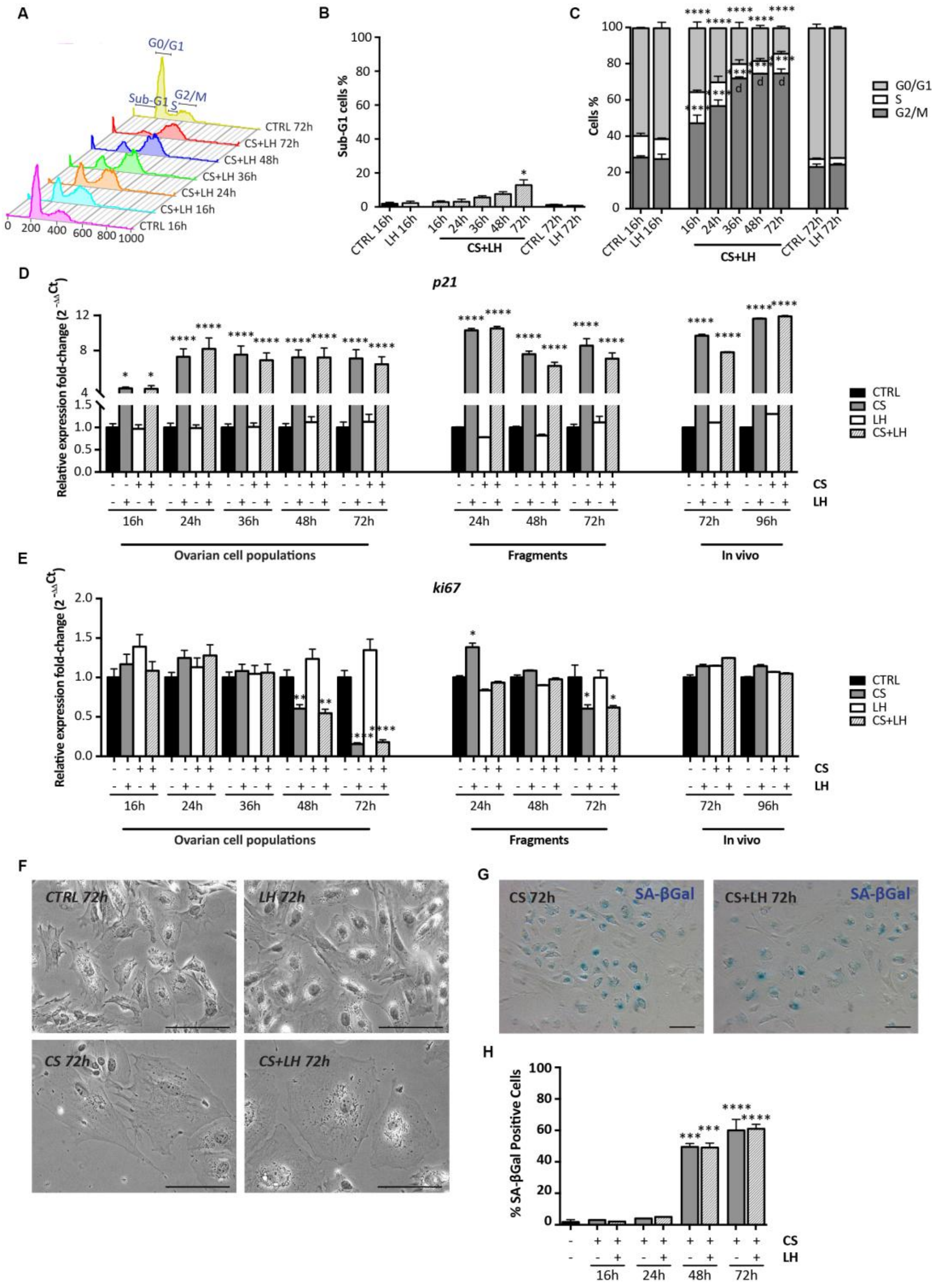

Supplementary Figure 7. LH has no effect on SIPS induced by CS in the ovarian somatic cells. (**A**–**C**) Representative flow cytometry of ovarian somatic cells treated with 10 μM CS with/out 200mIU/mL LH. The graph reports result as mean ± SEM of three experiments. Statistical differences *vs* control *p<0.05 ****p<0.0001. Statistical differences *vs* 16hrs in the G2/M phase *d* = p<0.0001. (**D**, **E**) Comparison of qRT-PCR for (**D**) p21 and (**E**) ki67 between *in vitro* (ovarian somatic cells and ovarian fragments) and *in vivo* (ovaries from intraperitoneal injected mice) conditions. Data are shown as mean ± SEM of three experiments. Statistical differences *versus* control group *p<0.05 **p<0.01 ****p<0.0001. (**F**) Ovarian somatic cells acquiring large and flattened morphology typical of SIPS after 72 hrs of culture either in the presence either of CS and CS+LH, scale bar = 100μm. (**G**) Similar SA-βgal activity in the same groups, scale bar =100μm. (**H**) The graph reports the percent of cells showing SA-βgal activity at the indicated times. Data are expressed as mean ± SEM of three experiments. Statistical differences *vs* control ***p<0.001 ****p<0.0001.

A B

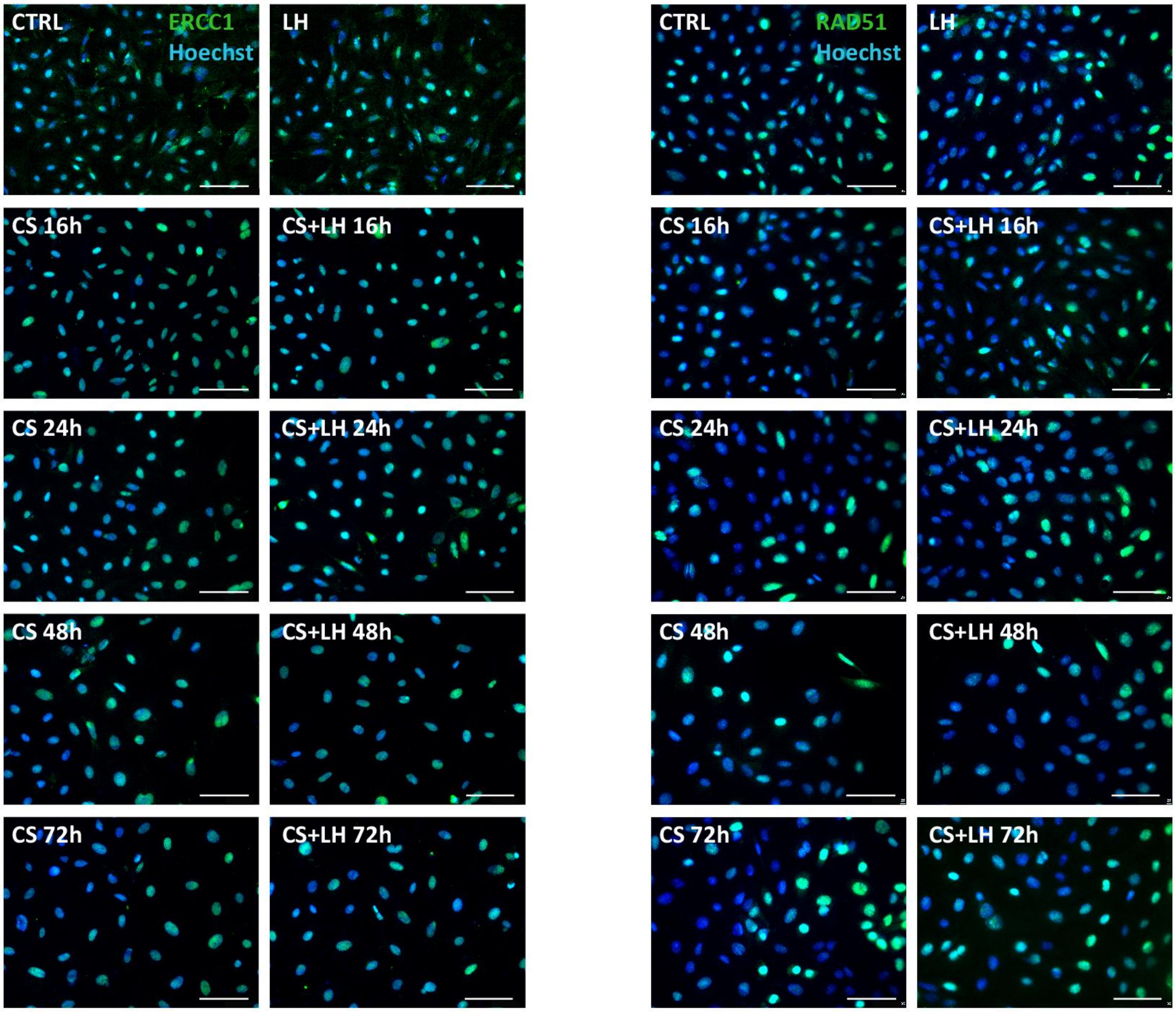

Supplementary Figure 8. Analysis of ERCC1 and RAD51 expression after treatment with/out CS and LH. (**A**, **B**) Representative IF of cells stained with ERCC1 (green, **A**) or RAD51 (green, **B**) after 16, 24, 48 and 72 hrs of treatment with 10 μM CS and with/out 200 mIU/mL LH Scale bar = 100 μm.
